# Interplay of active processes modulates tension and drives phase transition in self-renewing, motor-driven cytoskeletal networks

**DOI:** 10.1038/ncomms10323

**Published:** 2016-01-08

**Authors:** Michael Mak, Muhammad H. Zaman, Roger D. Kamm, Taeyoon Kim

**Affiliations:** 1 Department of Mechanical Engineering, Massachusetts Institute of Technology, 77 Massachusetts Avenue, Cambridge, Massachusetts 02139, USA; 2 Department of Biomedical Engineering, Boston University, 44 Cummington Mall, Boston, Massachusetts 02215, USA; 3 Howard Hughes Medical Institute, Boston University, Boston, Massachusetts 02215, USA; 4 Department of Biological Engineering, Massachusetts Institute of Technology, 77 Massachusetts Avenue, Cambridge, Massachusetts 02139, USA; 5 Weldon School of Biomedical Engineering, Purdue University, 206 S. Martin Jischke Drive, West Lafayette, Indiana 47907, USA

## Abstract

The actin cytoskeleton—a complex, nonequilibrium network consisting of filaments, actin-crosslinking proteins (ACPs) and motors—confers cell structure and functionality, from migration to morphogenesis. While the core components are recognized, much less is understood about the behaviour of the integrated, disordered and internally active system with interdependent mechano-chemical component properties. Here we use a Brownian dynamics model that incorporates key and realistic features—specifically actin turnover, ACP (un)binding and motor walking—to reveal the nature and underlying regulatory mechanisms of overarching cytoskeletal states. We generate multi-dimensional maps that show the ratio in activity of these microscopic elements determines diverse global stress profiles and the induction of nonequilibrium morphological phase transition from homogeneous to aggregated networks. In particular, actin turnover dynamics plays a prominent role in tuning stress levels and stabilizing homogeneous morphologies in crosslinked, motor-driven networks. The consequence is versatile functionality, from dynamic steady-state prestress to large, pulsed constrictions.

## Abstract (web summary)

The actin cytoskeleton is a complex network of filaments, cross-linking proteins and motors; although the components are recognised, the behaviour of the network is less understood. Here Mak *et al.* use a Brownian dynamics model that reveals actin turnover dynamics as a key regulatory mechanism controlling cytoskeletal states.

## Graphical abstract

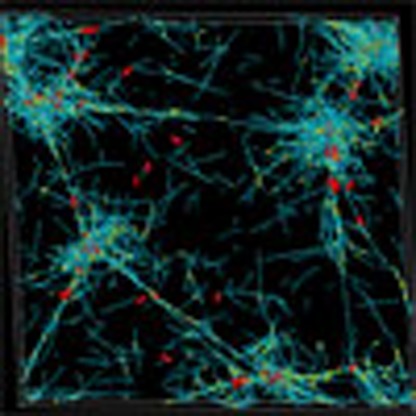

Biological systems require dynamic signalling and coordination of a multitude of biomolecules to generate complex phenomena and perform vital functions, from the migration of a single cell to the development of an organism^1^,^2^,^3^. While many interconnected cell signalling maps have been elucidated, much less is known about how biochemical reaction kinetics physically coordinate with mechanical components of cells to produce mechanical functions and phenotypes at the cytoskeletal network level. Two prominent examples are cancer metastasis and embryonic development. In cancer, cell deformability and force generation appear to be enhanced in aggressive, metastatic cells^4^,^5^,^6^,^7^,^8^. Tension enables cells to reorganize the extracellular matrix (ECM) and is correlated with increased invasiveness^4^,^6^,^9^. As cancer cells navigate through small pores in the extracellular matrix or across tight junctions in the endothelium during intra- and extravasation, they undergo large deformations and morphological changes^10^,^11^,^12^. Cancer cells may also exhibit increased active cytoplasmic fluctuations and transport^13^, which could have implications in signalling and metabolism^14^,^15^. Furthermore, during embryogenesis in a variety of organisms, including *Caenorhabditis elegans*, *Drosophila* and *Xenopus*, actomyosin foci are reversibly formed from an initially diffuse background in a transient manner, contributing crucially to intra-embryo constrictions, flow and polarity^16^,^17^,^18^. Disruption of actin dynamics leads to failed or ineffective apical constriction during *Drosophila* gastrulation, thus preventing proper tissue reorganization for ventral furrow formation^18^. Pulsations in foci formation and contractions are also prominent in various regions of the embryo, including the germ-band, mesoderm and amnioserosa in *Drosophila*^16^, suggesting universal mechanical patterns in development. Even when cells are in steady-state conditions, the cytoskeleton is highly dynamic, with time-variant molecular motor activity, actin turnover and active remodelling^19^,^20^. The underlying mechano-chemical interactions driving these diverse system-level characteristics are not clear^21^, but they likely involve a coordinated modulation of the mechanobiological circuitry, from adhesions and contractile motors to structural dynamics.

The mechanical functionalities of cells are conferred by the overarching states and capabilities of a network of cytoskeletal proteins. Complexity and diversity arise in part because the behaviour of the network is determined not only by the concentration or function of any single protein, but also by the relative activity of other complementary components, with interdependent reaction kinetics and mechanical properties. Recent studies using reconstituted active gels with actin, actin-crosslinking proteins (ACPs) and motors activated by ATP revealed the complex network remodelling dynamics due to internal force generation^22^,^23^,^24^,^25^,^26^,^27^,^28^. Active motors can contract actin gels, leading to network coarsening, tearing and local or global condensation, along with static or anomalous flow patterns—all dependent on relative ACP concentrations. While the findings have provided important insights into network motions and cluster size distributions, the parameter space in a biological system and multi-component network, even if oversimplified into reconstituted gels, is large and difficult to fully control and key physiological traits are not readily reproducible. For example, actin polymerization dynamics and turnover are typically neglected because of the complex regulation of various actin nucleators and modifiers^29^,^30^,^31^.

Recent mathematical models of actomyosin dynamics during development have started to include the turnover of components^32^,^33^,^34^. For instance, Dierkes *et al.*^32^ modelled actomyosin networks as contractile elements that follow a specified dynamic equation. These elements are composed of constituents, abstract force-producing molecules, that undergo exchange with a reservoir with specified on and off rates. A phase diagram can be derived that shows regions where the mechano-chemical oscillator collapses, oscillates or is stable, analogous to pulsatile contractile events during *Drosophila* development. Hannezo *et al.*^34^ developed a mathematical model that considers actin turnover and diffusion and determined a phase diagram of cytoskeletal states, including homogeneous states, stationary spatial patterns of actin that mimicked periodically spaced supracellular actin rings in the *Drosophila* tracheal tubule, and chaotic patterns that vary in time due to nonlinearity in actin turnover. While these models are relatively simple and general, it is unclear how different abstract components and coarsened terms of constitutive equations relate specifically to mechano-chemical kinetics and turnover mechanisms and dynamics of realistic cytoskeletal features, such as motor walking, actin treadmilling and ACP unbinding, which interact in large numbers.

In this study, we explore the direct connections between biochemical kinetics and mechanics at the cytoskeletal component level and simulate the resulting global cytoskeletal network. We use a three-dimensional (3D) Brownian dynamics computational model of the active actin cytoskeleton, incorporating core microscopic components and functionality—actin filaments that (de)polymerize, ACPs that bind and unbind in a force-dependent manner, and myosin II motors that walk and generate tension along actin filaments. These components are also mechanical in nature, with bending and extensional stiffnesses, enabling mechanics and kinetics to be concurrently simulated from the component to network scales. Our findings reveal that integrated mechanics and kinetics at the molecular level can produce and modulate a variety of cytoskeletal network level phenomena, including intracellular stress profiles and magnitudes, morphological states and stress fluctuations—all of which play important roles in physiological functions, from traction force production and intracellular transport to cell migration and morphogenesis. In particular, actin turnover dynamics, a prominent feature often overlooked in studies concerning intracellular contractile forces and network morphology, is a critical factor in regulating the mechanical states and phase transition of the cytoskeletal network—from homogeneous to clustered patterns. We further develop a simplified analytical and conceptual model—the active kinetic spring—that captures different cytoskeletal states as a function of system mechanics and kinetics. Last, we experimentally probe the cytoskeletal dynamics of live cells under disrupted actin polymerization, and we capture the time scales of morphological phase transition.

## Results and Discussion

### Brownian dynamics simulations of active actin networks

We perform computer simulations using a previously validated Brownian dynamics model of networks consisting of actin filaments, ACPs and motors^35^,^36^,^37^,^38^,^39^. A number of realistic parameters are incorporated to mimic the active cytoskeleton. Briefly, semi-flexible actin filaments are polymerized in the presence of ACPs and motors to form a well-connected, disordered network. ACPs bind actin dynamically and unbind in a force-dependent manner^40^ following Bell’s equation^41^. Motors are modelled after myosin II thick filaments (TFs), consisting of a backbone with multiple arms representing multiple myosin heads, enabling processive motors that can bind to multiple actin filaments and walk towards their barbed ends, generating internal tension in the network. Motor kinetics (walking, binding and unbinding rates) are governed by the parallel cluster model developed for calculating force-sensitive walking and unbinding rates of myosin thick filaments^42^. All of these components are also mechanical, with bending and extensional stiffnesses that provide elastic potential and structure. A homogeneous network is formed initially prior to motor walking and network contractions. An extended description of the model is in Supplementary Note 1 and a list of parameter values is in Supplementary Table 1.

In our model, actin is nucleated and polymerized into filaments and also depolymerized to monomers with given rates, reaching a dynamic steady state. Among various modes of actin turnover, we implemented actin treadmilling by allowing polymerization only at barbed ends and depolymerization only at pointed ends with similar rates. Additional details about the treadmilling model can be found in our recent study^39^. It was assumed that as an actin filament depolymerizes, bound ACPs and motors become unbound spontaneously. For the remainder of this paper including figures, we will use the term actin turnover rate to refer exclusively to the steady-state actin polymerization/depolymerization/treadmilling rate (per filament), as we do not explore other modes of actin turnover here. In this study, we emphasize on tuning actin turnover dynamics in the presence of actively walking motors. We performed simulations in domains that mimic 3D networks (3 × 3 × 3 μm with periodic boundary conditions in all directions) and domains that mimic 2D networks (8 × 8 × 0.5 μm with periodic boundary conditions only in the *x* and *y* directions). Unless specified otherwise, we simulated networks with an actin concentration of 25 μM and various indicated ratios of ACPs and motors to actin. In 2D networks, filaments typically lie along the planar (*x–y*) directions due to the shallow depth (*z*) of the domain, giving rise to morphologies that resemble the cytoskeleton of cells cultured on 2D substrates, as seen in super-resolution images^43^, as well as the cell cortex, which is typically a relatively thin shell on the order of several hundred nanometres thick. Internal stress profiles of each network were calculated by summing the tension acting on constituents crossing a cross-section divided by its area and then averaging over multiple cross-sections throughout the domain. Here we focus primarily on 3D networks, and thus unless specified, results were obtained in simulations of the 3D domain. However, we invoke 2D results in our analysis as well due to larger length scales probed and consideration of a different geometry. Furthermore, many prominent biological features are conferred at least partially by the cortical region, including high stiffness in the cell periphery^13^ and asymmetric constrictions in development^18^.

### Mechanical phase transition of active actin networks

**Figure 1:**
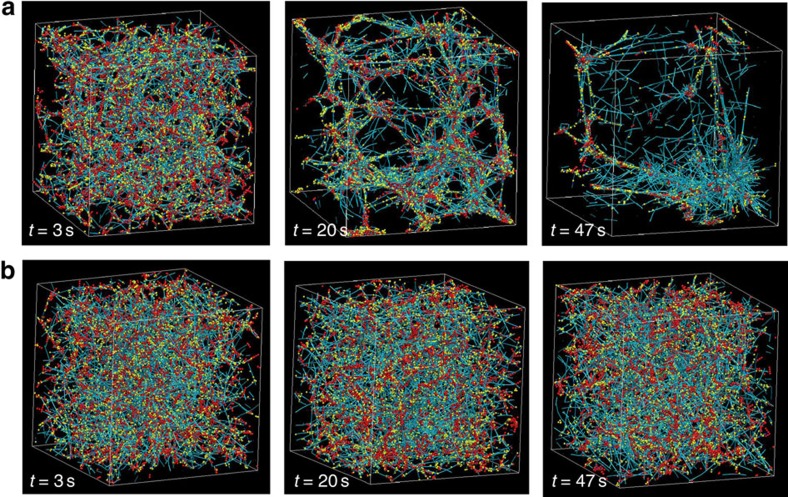
Network morphologies in the aggregated and homogeneous phases. (**a**) A 3D cytoskeletal network (3 × 3 × 3 μm) with an actin turnover rate of 30 s^−1^ with 25 μM actin, 5% ACPs (to actin) and 5% motors (to actin) at different time points. Motors start walking at *t=*0 s, and at *t=*3 s, the network is homogeneous. At *t=*20 s and 47 s, the network is progressively aggregating due to internal stress. (**b**) A network that has an actin turnover rate of 300 s^−1^ with 25 μM actin, 5% ACPs and 5% motors stays homogeneous and can sustain the motor-generated internal stress. Similar morphological states can be observed in 2D cortical networks (Supplementary Fig. 1). Teal, yellow and red are actin filaments, ACPs and motors, respectively.

Our study explored the mechanics and dynamics of crosslinked actin networks with the addition of two fundamental active processes—motor walking and actin turnover. These two processes confer onto an otherwise passive fibrillar gel, commonly studied using reconstituted actin networks and ACPs and characterized based on viscoelastic models^44^,^45^, active properties. Active matter is a more complex and dynamic class of material of which fundamental characterization is not fully developed. Our results showed that motor-driven and crosslinked actin networks exhibit two morphological phases—homogeneous networks with steady-state internal stresses and aggregated networks with unstable stresses that peak and diminish (Fig. 1, Supplementary Fig. 1, Supplementary Movies 1 and 2). This is consistent with experiments of reconstituted actomyosin networks that conglomerate into clusters over time when motors are active^22^,^24^. Such aggregates are not commonly seen in live cells under steady conditions, as cells typically exhibit relatively stable internal stresses^46^ with homogeneous networks, separated into the cortex and cytosol. However, during specific physiological events such as *Drosophila* gastrulation, actomyosin foci are dynamically generated and dissipated intermittently leading to large constrictions^47^. In our simulations, enhanced tension is exhibited in regions connecting different clusters (Supplementary Fig. 2), potentially indicating the long-range coherence necessary for cooperative contractions and cytoskeletal flows. The fundamental drivers of this morphological phase transition are not well-understood. Therefore, we investigated the role of several fundamental mechanical regulators of the cytoskeleton—namely actin turnover via polymerization dynamics (treadmilling), ACPs and motor activity.

**Figure 2:**
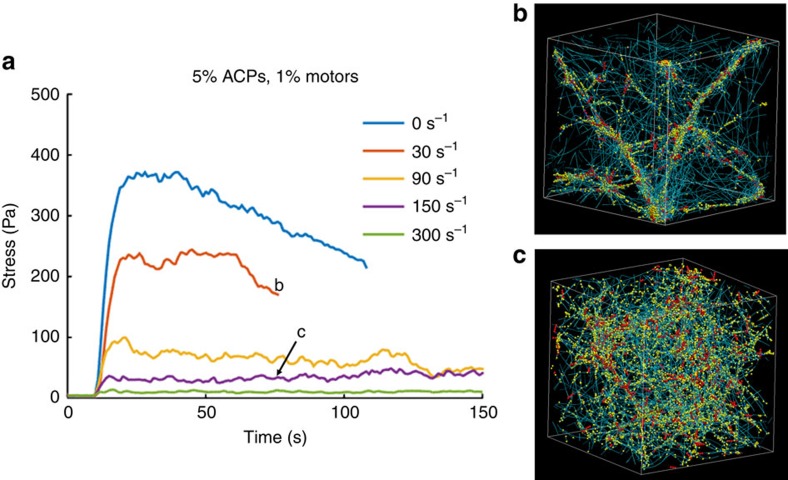
Time evolution of internal stresses. (**a**) Typical stress versus time profiles in simulated cytoskeletal networks with active motors, actin turnover and ACP binding dynamics. These results are from 3D networks with 25 μM actin, 1% motors and 5% ACPs at the specified actin turnover rates (legend). At low turnover rates, the stress peaks and then dissipates corresponding to network aggregation, whereas at high turnover rates the stress is lower but remains stable, indicative of a relatively homogenous network. **b**,**c** show the network morphologies in the corresponding time and configuration indicated in **a**. Teal, yellow and red are actin filaments, ACPs and motors, respectively.

Our results demonstrate that actin turnover can enable cytoskeletal networks to avoid aggregation and sustain steady-state internal stresses. Stress profiles of these networks depend on the actin turnover rate, in addition to ACP and motor concentrations (Fig. 2 and Supplementary Figs 3 and 4). For unstable networks that collapse due to motor-induced forces, the stress peaks at a relatively high value, followed by dissipation, during which ACPs unbind and stress is relieved. These networks become aggregated over time. Note that high network aggregation has extremely high computational cost and thus those simulations are stopped at earlier time points. By contrast, for stable networks, the stress increases initially as motors walk and reaches a steady-state level while the network maintains a homogenous morphology. For an actin network configuration with fixed concentrations of actin, motors and ACPs, there exists a critical range of actin turnover rates below which the network collapses into aggregates with no sustained global stresses and above which the network is homogeneous with sustained connectivity and internal stress (Figs 1 and 2). Moreover, as the concentration of ACPs increases relative to the concentration of motors, the critical turnover rate decreases. The load generated by the motors is distributed over more ACPs, resulting in a reduced force-dependent unbinding rate per ACP, which adds stability to the network. As the ratio of ACPs to motors reaches ∼100, the critical rate approaches 0. For a fixed concentration of ACPs and actin, a higher motor concentration increases the critical actin turnover rate, since the internal load in the network is increased. Furthermore, in cortical actin geometries in which we can simulate larger length scales at reasonable computational cost, increasing actin filament length and concentration increases the internal stress and the stability of the network (Supplementary Fig. 5). Increasing ACP concentration has been shown experimentally in reconstituted actomyosin networks to increase network percolation, leading to a transition from local to global contractions beyond a critically connected state^24^. Longer actin filaments may play a similar role in enhancing network connectivity.

**Figure 3:**
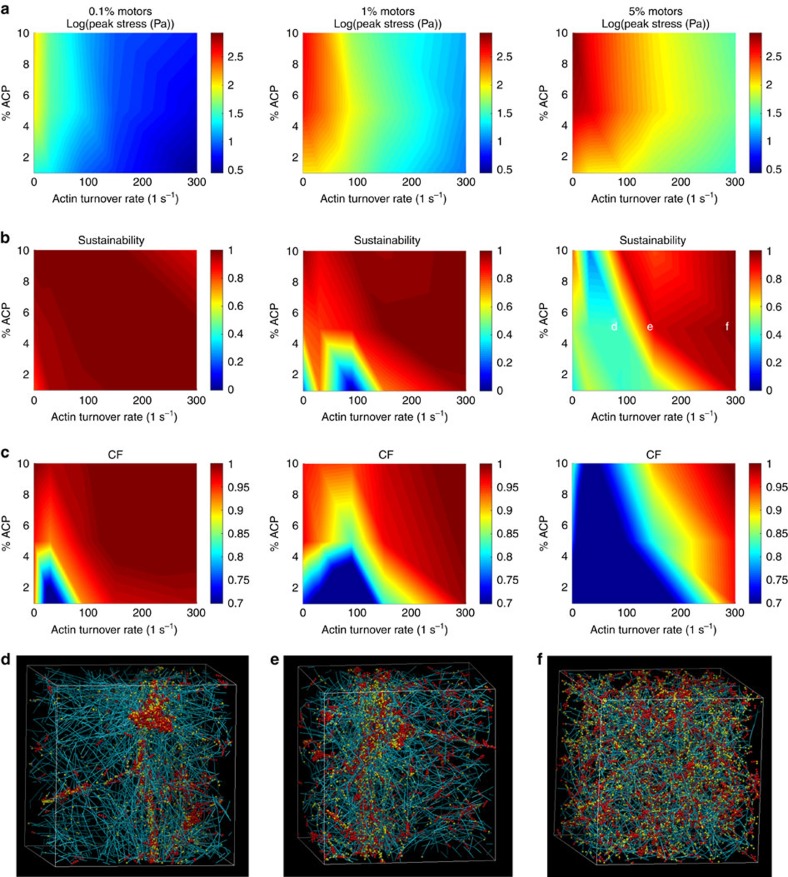
Mechanical phase transition of 3D cytoskeletal networks. (**a**) The peak stresses, (**b**) stress sustainability factor (see Supplementary Note 2) and (**c**) clustering factor CF (see Supplementary Note 3) at *t=*40 s after motor activation are plotted as a function of the actin turnover rate and % ACP. (See Supplementary Fig. 8 for CF heat maps at the end of simulation time). Each column represents a different motor concentration in the network. The actin concentration in these simulations is 25 μM. The peak stress (maximum stress exhibited by the network over time once the motors are activated averaged over a 10-s time interval) is reduced with increasing actin turnover rate and is enhanced with increasing motor concentration. The sustainability of the generated stresses and homogeneous network morphology tend to scale proportionately with ACP concentration and actin turnover rate and inversely with motor concentration. (**d**–**f**) Representative network morphologies of the indicated points in **b** 51 s after motor activation. Teal, yellow and red are actin filaments, ACPs and motors, respectively.

Figure 3a shows the peak stress, defined as the maximum stress exerted by the network, as a function of actin turnover rate and concentrations of ACPs and motors. Typical average traction stresses of cells on 2D substrates, as measured by traction force microscopy, are on the order of 100 and 1000 Pa (refs 6, 48), comparable to reconstituted actomyosin gels and cytoplasmic extracts^25^, though there are variations throughout the cell and dependence on factors such as substrate stiffness, spread area, geometry and cell type. In our simulations, higher stresses can be achieved by simulating higher molecular concentrations at the expense of high computational cost. Our results show that the peak stress decays with increasing actin turnover rate and rises with increasing motor concentration. To quantify whether the generated stress can be sustained in steady-state (i.e., stable, non-decaying average stress over long time scales as opposed to stress profiles that peak transiently in the beginning before decay) and whether the network is able to maintain a homogeneous morphology or aggregate, we devise two metrics termed the relative sustainability factor, or sustainability (*S*; see Supplementary Note 2 and Supplementary Fig. 6) and the clustering factor (CF; see Supplementary Note 3 and Supplementary Fig. 7). When *S*∼1, the network is able to sustain stresses over long periods, whereas when *S*∼0 stresses diminish rapidly over time. The rate of stress collapse is proportional to 1−*S*. Similarly, when the network morphology is homogeneous CF∼1, and when the network morphology is highly aggregated CF∼0. *S* and CF therefore enable us to quantitatively assess the mechanical state of the network and the onset of phase transition from stress-sustaining homogeneous networks to aggregated networks that build and subsequently diminish internal stresses. In Fig. 3b,c (and Supplementary Fig. 8), *S* and CF are mapped as a function of ACP concentration, motor concentration and actin turnover rate, juxtaposed below the peak stress maps of the same network configurations in Fig. 3a. Representative network morphologies of the corresponding peak stress, *S*, and CF are shown in Fig. 3d–f. This illustrates the overall mechanical state of the cytoskeletal network based on the interplay between active and passive mechano-chemical components.

Through our simulations, we demonstrated and elucidated the details of the transition from relatively uniform to condensed networks under tension. As the ACP concentration is increased, each ACP bears less stress and its unbinding rate is reduced. We further showed that actin turnover, which is a dynamic process ubiquitous in *in vivo* biomechanical processes, plays an important role in regulating the mechanical state of cytoskeletal networks, in addition to the concentration effects of different molecular species. This has not been considered in recent experiments, as actin filaments in reconstituted systems are typically stabilized, that is, no continuous (de)polymerization or nucleation.

The phase maps of *S* and CF demonstrate the different possible regimes of stress profiles and network morphologies, respectively, as a function of actin turnover, ACP concentration and motor activity. They indicate transition regimes where the cytoskeleton changes phases and how modulation in three fundamental cytoskeletal components can lead to drastic changes in the form and functionality of active intracellular networks. The metrics *S* and CF can both be measured experimentally using traction force microscopy and fluorescence imaging of actin, respectively. Recent studies in *Drosophila* embryos showed that myosin activity and actin turnover are both necessary for periodic phase transitions in morphological and contractile states during large constriction events^18^,^49^, corroborating the multiple axes driving transitions indicated in our phase maps, which suggest potential positive feedback mechanisms or robustness via redundancy.

### Interplay between actin turnover and motor walking rate

We further explored the interplay between different kinetic parameters in regulating the mechanical state of the cytoskeleton. Signalling cascades and coordinated biological phenomena, such as migration and morphogenesis, often require the modulation of signalling proteins such as Rho GTPases or actin nucleators, which control the kinetic rates regulating motor activity and actin turnover. For stable networks above a critical turnover rate, continued increase in turnover rate, while maintaining stable networks, diminishes the magnitude of the sustained internal stresses (Figs 2 and 3 and Supplementary Figs 3 and 4). At very high turnover rates, the internal stress is close to 0. The cause of this phenomenon is as follows. As motors bind to and walk towards the barbed ends (which are the polymerizing ends) of anti-parallel filaments, they build up stress within the actin network by pulling and inducing strain in the filaments. However, the stress requires time to build up and as the motors walk farther along the filaments, larger stresses are generated. As the actin turnover rate is increased, filaments disintegrate more quickly as motors are walking, causing motors and ACPs to unbind and stress to be relaxed. At steady state, less stress is generated because motors on average have walked a shorter distance before unbinding events relieve internal tension.

To validate and explore these dynamics in more detail, we varied the motor walking rates from half to twofold. Changing ATP concentrations or activating signalling proteins such as RhoA could potentially accomplish this^50^. Faster motor walking will build up internal stress faster. We found that a constant ratio of the actin turnover rate to the motor walking rate produces similar stress and morphological profiles, at least for higher turnover rates. For instance, for networks in the homogeneous phase, doubling or halving both rates will result in similar peak stresses as if both rates were not changed, (Supplementary Fig. 9), where the peak stress versus turnover rate curves collapse to a master curve when the turnover rate is scaled by the relative motor walking rate. In addition, the ratio of these rates controls the mechanical phases, since it modulates the peak stresses of the networks, which determine whether or not the network can remain stable.

### Reversible formation of actomyosin foci

**Figure 4:**
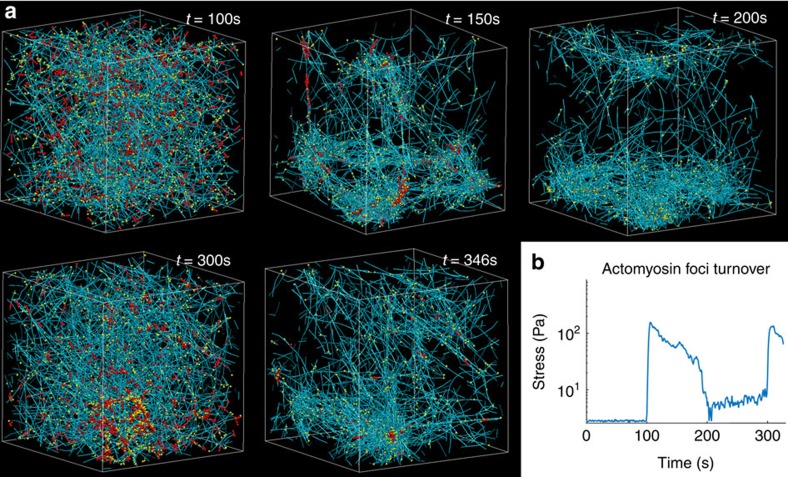
Reversibility of actin aggregation in the active cytoskeleton. This network consists of 25 μM actin, 5% ACPs, 1% motors and an actin turnover rate of 300 s^−1^. (**a**) At *t=*100 s, motors are activated and actin turnover is disabled, leading to aggregation over time. At *t=*200 s, actin turnover is enabled, leading to foci disintegration and the regeneration of a homogeneous network. At *t=*300 s, actin turnover is suppressed again, enabling foci to reform. (**b**) The stress versus time profile for the network in **a**, displaying the relation of foci formation and turnover to stress. Foci formation leads to large but transient stress build-up. Actin turnover enables the network to regenerate and allows for subsequent stress build-up, enhancing contractile behaviour over longer periods. This suggests the driving factors and functional implications of pulsatile actomyosin contractions and foci formation during embryogenesis. In these simulations, we used larger motors with 256 heads (rather than 64) which appear to be able to aggregate more crosslinked networks more quickly. Teal, yellow and red are actin filaments, ACPs and motors, respectively.

During various stages of development across a number of species, actomyosin foci formation and disintegration are observed and suggested to be important in large-scaled contractions and tissue reorganization^16^. For example, *Drosophila* gastrulation entails pulsatile actomyosin foci formation and corresponding large transient contractions, which lead to apical constriction and tissue invagination^47^. However, the functional implications of these temporal characteristics and network morphologies are not clear. The intricate rebalancing of mechanics and reaction kinetics leading to the activation and deactivation of these phase separation events is also not well-elucidated. Our results suggest that foci formation, due to either a reduction in actin turnover rate or increase in motor activity across the phase transition regime, leads to a drastic but transient increase in internal stress levels, which enhances large-scale contractions. However, over time as the cytoskeleton aggregates, network connectivity is lost, resulting in a reduction in contractility and disconnected foci. To continue contracting globally, the network must regenerate and reconnect via actin turnover (the actin turnover to motor activity ratio must increase) and then re-activate the foci formation phase (reduction in the aforementioned ratio)—thus requiring pulsatile behaviour. These stages are simulated in Fig. 4 and Supplementary Movie 3. The contractile pulses seen *in vivo* are on the order of 100 s (refs 16, 47, 51). Thus, we have chosen this time scale to pulse changes in actin turnover rates in our simulations (Fig. 4). Interestingly, over this time scale, the internal stress profile at low actin turnover peaks and then substantially diminishes. This suggests that network recovery via increased actin turnover or decreased myosin activity is required after large contractions lasting ∼100 s for further contractions to occur. It also appears that ∼100 s of recovery time can enable the next constriction event to reach a comparable magnitude in stress as the previous. This, in addition to a ratcheting mechanism likely involving cell shape stabilizing actin cables^18^,^51^, may lead to sustained contractile activity. Interestingly, according to our simulations as shown in Fig. 2b, actomyosin cable-like structures and possibly other superstructures beyond spherical aggregates may be spontaneously generated, especially near the phase transition regime. In particular, our results highlight the importance of actin turnover and network regeneration in controlling mechanical state transitions. Furthermore, foci formation conglomerates cellular content and, without network regeneration, minimizes network connectivity. This facilitates intracellular transport and asymmetric redistribution of content through flow, as observed in *C. elegans* embryogenesis before pseudocleavage^17^. If the network were well-connected and elastic rather than fluid, restoring forces would make large-scale transport of cytoskeletal constituents more difficult.

While the kinetics and molecular regulation of pulsatile behaviour during development are not well-known, it has been shown that actin assembly regulators such as the formin Diaphanous and myosin activators such as RhoA are both required for efficient contractility during apical constriction^52^. One potential mechanism for how this pulsatile behaviour is regulated *in vivo* is through negative feedback, whereby contractile actomyosin networks recruit aPKC, which diminishes contractility^52^. Another possibility found experimentally is that Rho-associated kinase (ROCK), which regulates myosin II phosphorylation, activates myosin in a pulsatile manner^49^. ROCK aggregation coincides with myosin aggregation, suggesting that the phosphorylation of myosin is pulsed leading to pulsatile actomyosin foci formation and contractions. Phosphomimetic mutants did not show pronounced pulsations, but instead exhibited more continuous clustering of myosin and reduced rapid constrictions. In addition, ROCK indirectly reduces actin turnover by activating LIM kinase which deactivates ADF/cofilin^53^, suggesting that actin turnover may also be pulsed. Preventing actin polymerization via Cytochalasin D inhibits pulsatile behaviour, inducing *Drosophila* embryos to form myosin aggregates that do not continuously coalesce^47^ and to generate disconnected medioapical actomyosin networks that fail to promote apical constriction^18^. These studies suggest that actin turnover dynamics and regulation of motor activity are both required for pulsatile constrictions during development in *Drosophila*. Our simulation results and heat maps for *S* and CF provide quantitative data towards how the mechanical phase transition can occur and what ratios in activity levels of actin turnover and myosin contractility are required for foci assembly and disassembly. In particular, from a homogeneous state, decreased actin turnover and enhanced motor activity both lead towards cluster formation, suggesting synergistic signalling axes via ROCK.

### Higher order mechanical features of intracellular networks

Active cytoskeletal networks exhibit additional properties that play important roles in cell function. In cells, there exist many factors that can alter binding and turnover kinetics. Different ACPs can have different dissociation rates and bond behaviours, such as catch bonds that have decreased unbinding rates under tension^54^. We simulated the limiting case of permanent bonds, as exhibited by fascins^55^ and scruins^45^, that do not unbind intrinsically and found that actin turnover can assume dual roles in destabilizing and sustaining internal stresses (Supplementary Fig. 10). Furthermore, actin filaments can bend and sever due to myosin contractility and ADF/cofilin, leading to enhanced actin turnover. Severing can alter stress profiles and diminish stress magnitudes (Supplementary Fig. 11). See Supplementary Note 4 for more details on these additional modulators of cytoskeletal kinetics.

**Figure 5:**
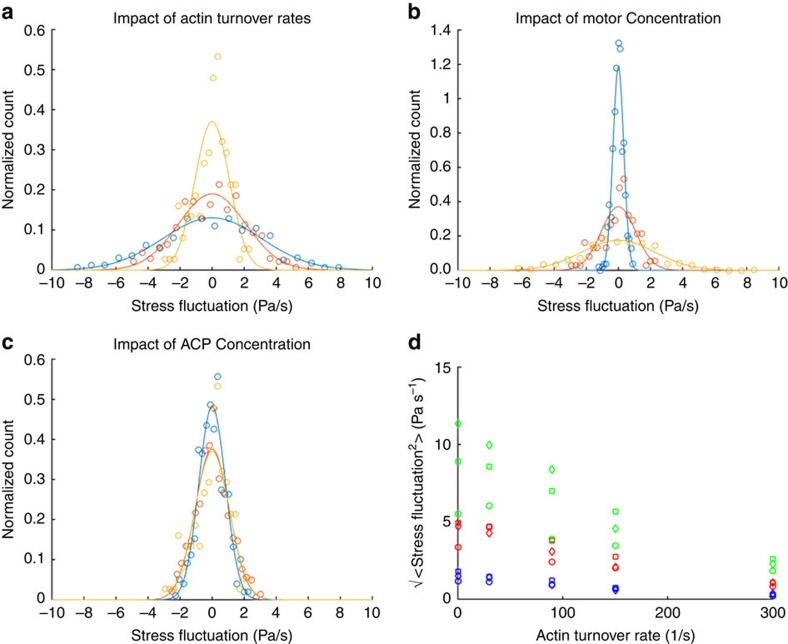
Stress fluctuation magnitudes are modulated by network turnover and motor activity. (**a**) Network configuration (10% ACPs, 1% motors). Increasing the actin turnover rate from 90 (blue) to 150 (red) to 300 s^−1^ (blue) leads to a decrease in the spread of the stress fluctuations. (**b**) Network configuration (10% ACPs, 300 s^−1^ actin turnover rate). Increasing the motor concentration from 0.1% (blue) to 1% (red) to 5% (yellow) increases the s.d. of the stress fluctuation distribution. (**c**) Network configuration (1% motors, 300 s^−1^ actin turnover rate). Increasing the ACP concentration from 1% (blue) to 5% (red) to 10% (yellow) does not appear to alter the s.d. of the stress fluctuation distribution. Stress fluctuations were measured over a total area of 27 μm^2^ (9 μm^2^ in each orthogonal axis) in each simulation domain. Stress fluctuation distributions from computational data (circles) were normalized and fitted to a normalized Gaussian with identical s.d. (lines). (**d**) Amplitude of stress fluctuations. Blue, red and green correspond to motor concentrations of 0.1%, 1% and 5%, respectively. Circles, squares and diamonds correspond to ACP concentrations of 1%, 5% and 10%, respectively. Percentages are relative to the actin concentration of 25 μM used in these simulations.

In addition to generating tension, molecular motors induce active stress fluctuations throughout the cytoskeleton, providing enhanced random but non-thermal motions in intracellular particles and organelles^13^. This may be important in intracellular mechanotransduction or redistribution of macromolecules in crowded environments (see Supplementary Note 5), and stress fluctuations are modulated by motor activity and actin turnover (Fig. 5).

### Active kinetic spring model

**Figure 6:**
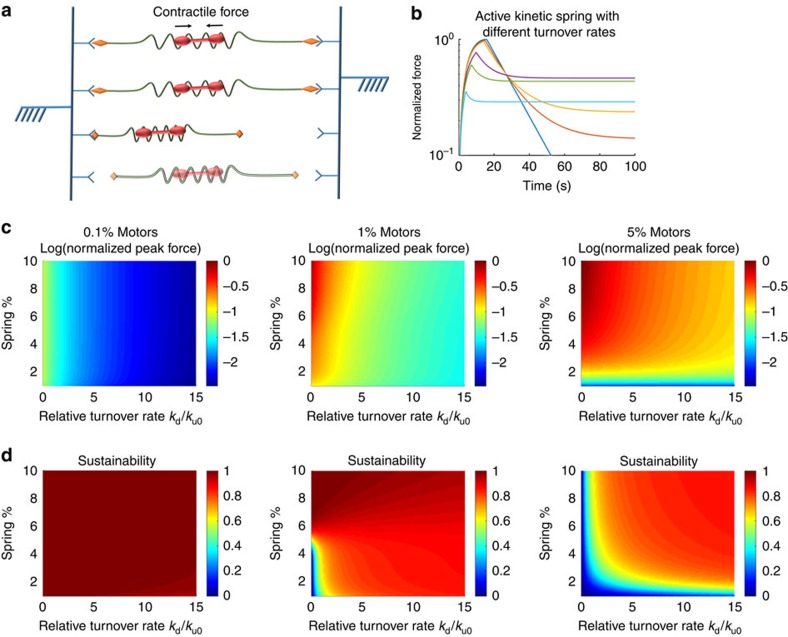
Active kinetic spring model. (**a**) Schematic of the model—cytoskeletal springs (green) are connected to fixed boundaries (blue) by kinetic elements (orange diamonds and Y shapes) that can bind and unbind at force-dependent rates. Myosin motors (red) actively contract the springs internally, generating force. Components can also appear or disappear via turnover (transparency). The total force on the walls is the sum of the contractile forces from bound springs. (**b**) Force profiles based on the model for 25 μM actin and 0.1 ratio of springs (that is, ACP-actin spring constructs) and 0.05 ratio of motors to actin at different network turnover rates. Profiles with transient peaks and stable forces are both observed, as consistent with Brownian dynamics simulations. Curves from top to bottom (based on peak force) represent network turnover rates *k*_d_ of 0, 0.1, 0.2, 1, 2 and 5 × relative to *k*_u0_. (**c**) Heat maps of the log of the normalized peak force for networks with different configurations. Forces are normalized to networks with 0.1:0.05 ratio between springs and motors and actin concentration of 25 μM. (**d**) Heat maps of *S* for corresponding configurations in **c**. The active kinetic spring model is able to qualitatively capture similar mechanical properties compared with Brownian dynamics simulations.

To synthesize our findings, here we develop a simplified conceptual model—the active kinetic spring. The purpose of this model is to produce simple constitutive relations at the cytoskeletal network scale that can capture qualitatively the impact of molecular-level properties as predicted by our Brownian dynamics simulations. We consider the actin-ACP construct to act as a spring that also exhibits force-sensitive binding kinetics and dynamic turnover (disintegration and regeneration). The effective spring constant of a system of these spring elements depends on the number of internal springs that are active, that is, the ACPs are bound to and connect different actin filaments. Unbound or disintegrated springs do not contribute to the net spring constant and cannot transmit forces through the system. Internal motors contract the springs to generate force across the system. A simplified schematic of the model is shown in Fig. 6a and a derivation of an analytical solution to this system, with some assumptions, is shown in Supplementary Note 6. The solution to this model is able to reproduce qualitatively similar force profiles and phase maps for peak forces and sustainability for different configurations, as shown in Fig. 6b–d, compared with Brownian dynamics simulations. The active kinetic spring model provides a simple means to assess the impact of various mechano-chemical features of the cytoskeletal machinery on the dynamic mechanical state of the global network.

### Experimental disruption of actin turnover dynamics

Previous studies have demonstrated that disrupting actin polymerization in cells using drugs such as Cytochalasins will generate intracellular actin aggregates and diminish cell tension^56^,^57^,^58^, which is consistent with our simulation results. Loss of connectivity would result in a reduction in the elasticity of the cytoskeletal network, consistent with experimental work showing increased fluid-like intracellular particle motion at relatively long time scales^59^. Furthermore, experiments with reconstituted networks of actin, ACPs and myosin motors are usually performed in (phalloidin) stabilized actin^23^ or without actin nucleators^24^,^60^, potentially inadvertently mimicking polymerization inhibitor treatment in cells. Again in those studies, localized aggregates are formed, unless if the actin crosslinking is high.

**Figure 7:**
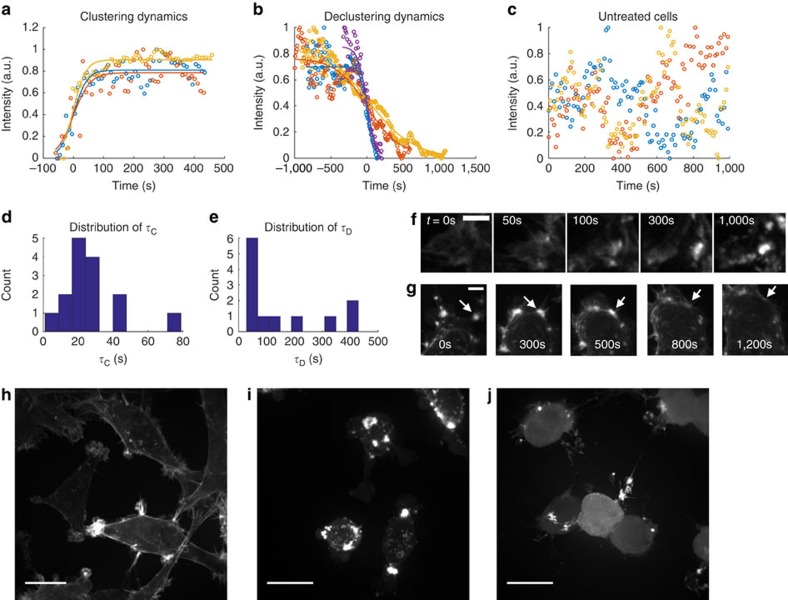
Clustering dynamics in living cells. (**a**) Representative data (open circles) and fits (curves) of the normalized intensity of fluorescent F-actin in cells during (**a**) clustering after Cytochalasin D treatment, (**b**) declustering after washing out Cytochalasin D and (**c**) normal conditions for untreated cells. For **a**,**b**, curves and data are shifted on the time axis by the time delay offset (see Methods) such that the fitted sigmoid is centred at *t=*0. The condition of the media was initially changed to Cytochalasin D or washed and replenished with growth media for **a**,**b**, respectively, at the first time point of each data set. Different colours represent different representative clusters or intracellular positions across multiple cells. Distributions of time constants for (**d**) clustering (average *τ*_C_=28±5 s (s.e.m.), *n*=15 clusters) and (**e**) declustering (average *τ*_D_=155±42 s (s.e.m.), *n*=12 clusters). Representative time evolution during cluster (**f**) formation and (**g**) disintegration inside live cells. In **f**,**g**, *t=*0 corresponds to the time when the drug is added or removed. Scale bars, 5 μm. Arrows point to the same cluster at different time points. Typical cells that are (**h**) untreated, (**i**) treated with Cytochalasin D for 1 h, and (**j**) treated with Latrunculin A for 1 h. Images in **h**–**j** are maximum projections from z-stacks with 1 μm z-resolution. Scale bars, 20 μm.

Here we performed experiments in modulating actin turnover in live cells expressing fluorescent F-actin. We measured actin clustering dynamics immediately after cell treatment with Cytochalasin D, which caps actin barbed ends and prevents polymerization and depolymerization there^57^. We found that actin aggregates after Cytochalasin D treatment and declusters after washout, indicating reversibility of actin clustering dynamics, and measured the time scales of clustering and declustering after disrupting or rescuing intrinsic actin polymerization dynamics (Fig. 7a,b). Untreated cells do not show these dynamics (Fig. 7c). Clustering and declustering dynamics were fit to simple sigmoid models to extract quantitative time constants (see Methods) and their distributions (Fig. 7d,e). On average, the time constant for cluster formation *τ*_C_ is 28 s while the time constant for declustering *τ*_D_ is 155 s, which are consistent with our computational results (on the order of 100 s). Representative time slices of the formation and disintegration of actin clusters are shown in Fig. 7f,g and Supplementary Movies 4 and 5. Latrunculin A is another actin disrupting molecule that functions by binding to actin monomers and preventing their association to actin filaments^61^. Interestingly, only Cytochalasin D treatment induces the formation of a multitude of actin clusters inside cells, whereas Latrunculin A-treated cells have more diffuse actin distributions with few dispersed clusters, even though in both cases overall cell morphologies are rounded (Fig. 7g–i). This may be the result of Latrunculins binding to actin monomers, which impacts polymerization but not depolymerization rates and leads to actin filaments disintegrating more quickly over time. Both Cytochalasins and Latrunculins have been shown to soften the cytoskeleton and reduce cell contractile forces^58^,^62^,^63^, but here we show that they have distinct effects on the cytoskeletal morphologies of living cells.

Our results empirically demonstrate that actin (de)polymerization dynamics are critical in regulating the morphological state of the active cytoskeleton and reveal the time scales of global morphological state transitions. They show that pulsing turnover rates is a possible mechanism in generating pulsatile actin foci formation, and the time scales are consistent with such pulsations during embryonic development as described earlier. Actin turnover, which is prominent in live cells and mediated by proteins such as Arp2/3 and formins in the cortex^19^, therefore must be considered to fully capture the *in vivo* mechanical states of cells. Furthermore, actin foci that form in living cells as a result of suppressing actin polymerization dynamics have been shown to co-localize with myosin and ACPs^64^. Myosin IIa plays a critical role in modulating the motion of these foci. Under normal conditions, the actin foci undergo dynamic drift-diffusion motion and can merge, whereas under suppressed myosin IIa activity via blebbistatin treatment or knockdown the foci exhibit suppressed motion that appears only diffusive.

## Conclusions

Using Brownian dynamics simulations, we demonstrated important functions of universal intracellular features—actin turnover, ACP unbinding dynamics and motor activity—in regulating the mechanical state of the cytoskeleton. We further elucidated novel fundamental roles of actin turnover dynamics in regulating internal tension magnitudes and fluctuations and morphological phase transition. Control and deregulation in these properties have implications in a wide range of biological phenomena, from intracellular transport and cell force generation to embryonic development and metastatic disease. Previously, actin polymerization has been implicated primarily in cell protrusions at the leading edge, but here we demonstrated its importance throughout the cell, thus serving as a potential multipurpose evolutionary tool to control multiple aspects of the intracellular mechanical state. Overall actin turnover confers self-renewability, myosin motors actuate contractility and ACPs enable local and global connectivity. These features constitute the cytoskeleton as active matter with diverse functionality that exhibits self-healing and self-destroying capabilities.

The phase maps generated here provide a means of understanding integrated network responsivity to perturbations across multiple tangible and pharmacologically targetable components in the mechanobiological circuit. The demarcation of phase transition regimes provides insights towards the regulation of sudden and transient mechanical behaviours in active biological systems, including tissue constrictions and network clustering. In particular, our results suggest a fundamental reason for why pulsation in clustering must occur during large constriction events in various embryos—high stresses in the cytoskeleton cannot be sustained as the network collapses and disconnects. Pulsing across phase transition boundaries enables the network to reinitialize and generate large stresses repeatedly over extended periods.

## Methods

### Cell culture and experiments

MDA-MB-231 cells expressing fluorescent actin filaments (LifeAct) were cultured at 37 °C, 5% CO_2_ with DMEM supplemented with 10% foetal bovine serum and 1% penicillin-streptomycin. Cells were a generous gift from the Lauffenburger Lab. These cells were used because they are highly active and contractile, features that we are interested in and are important in cancer progression^6^,^65^. During experiments cells were plated on 2D glass-bottom wells. After 1 day of incubation, cells were then treated with 2.5 μM Cytochalasin D (Sigma-Aldrich) or 1 μM Latrunculin A (Sigma-Aldrich). The concentrations of drugs were chosen such that their impact on cell mechanical properties is around maximal saturating levels^56^,^66^. Cells were imaged with a spinning disk confocal microscope using a × 63, 1.4 numerical aperture oil immersion objective (Leica). Fluorescent images were processed with ImageJ.

### Actin clustering experiments and analysis

For time lapse experiments with Cytochalasin D and Latrunculin A treatments, actin disrupting drugs were added and then z-stacks of fluorescent actin were acquired immediately thereafter at a time resolution of 10 s, z step size of 2.5 μm and *x–y* pixel size of 0.179 μm. For declustering experiments, the media containing drugs was removed after ∼1 h of treatment, and the sample was washed three times with growth media and imaged immediately afterwards with the same imaging scheme. Untreated cells were imaged in the same manner. To measure the dynamics of clustering and declustering, a box of 5.4 × 5.4 μm was generated on ImageJ and centred on regions with existing or emerging actin clusters. Maximum intensity z-projections of the images were used. The size of the box was chosen such that it encapsulates individual clusters to be measured. Average intensity was measured within the generated box region. The intensity measurements are then post-processed via custom Matlab codes to calculate the time scales of clustering and declustering. Raw data were first normalized by subtracting the minimum intensity and dividing by the maximum intensity of each measured cluster.

To calculate the timescale of clustering, normalized intensity measurements were fit to a simple sigmoid with a single time constant:




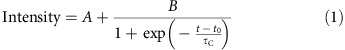




where *t* is time, *A* and *B* are fitting constants for the baseline and plateau of the normalized intensity curve, *t*_0_ is the time delay offset since not all clusters start clustering at the same time after drug treatment of cells, and *τ*_c_ is the clustering time constant, the key parameter of interest. Representative data and corresponding fits are shown in Fig. 7a. Our results show that *τ*_c_=28±5 s (*n*=15 clusters). *R*^2^ of the fit to the data is 0.77±0.05.

To calculate the timescale of declustering, normalized intensity measurements were fit to:




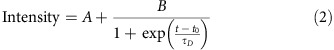




where the fitting parameters are the same as for the case of clustering except *τ*_D_ is the declustering time constant. Representative data and corresponding fits are shown in Fig. 7b. Our data show that *τ*_D_=155±42 s (*n*=15 clusters). *R*^2^ of the fit to the data is 0.83±0.03.

Representative data from untreated cells under the same imaging conditions are shown in Fig. 7c.

## Additional information

**How to cite this article:** Mak, M. *et al.* Interplay of active processes modulates tension and drives phase transition in self-renewing, motor-driven cytoskeletal networks. *Nat. Commun.* 7:10323 doi: 10.1038/ncomms10323 (2016).

## Supplementary Material

Supplementary Information Supplementary Figures 1-11, Supplementary Table 1 and Supplementary Notes 1-6

Supplementary Movie 1 Cytoskeletal network with 10% ACP, 5% motors, 25 μM actin, and an actin turnover rate of 30 s^-1^

Supplementary Movie 2 Cytoskeletal network with 10% ACP, 5% motors, 25 μM actin, and an actin turnover rate of 300 s^-1^

Supplementary Movie 3 Cytoskeletal network from Fig. 4 with pulsed actin turnover rates.

Supplementary Movie 4 MDA-MB-231 cells expressing fluorescent F-actin immediately after Cytochalasin D treatment. The video frame rate is 100x real time.

Supplementary Movie 5 MDA-MB-231 cells expressing fluorescent F-actin immediately after washing out Cytochalasin D. The video frame rate is 100x real time.
