# Supplementary material for: Interplay of active processes modulates tension and drives phase transition in self-renewing, motor-driven cytoskeletal networks
Source: Nat Commun. 2016 Jan 8;7:10323. doi: 10.1038/ncomms10323 (PMC4714927; doi:10.1038/ncomms10323)
Supplement: Supplementary Information — Supplementary Figures 1-11, Supplementary Table 1 and Supplementary Notes 1-6 [file ncomms10323-s1.pdf]

Supplementary Figure 1

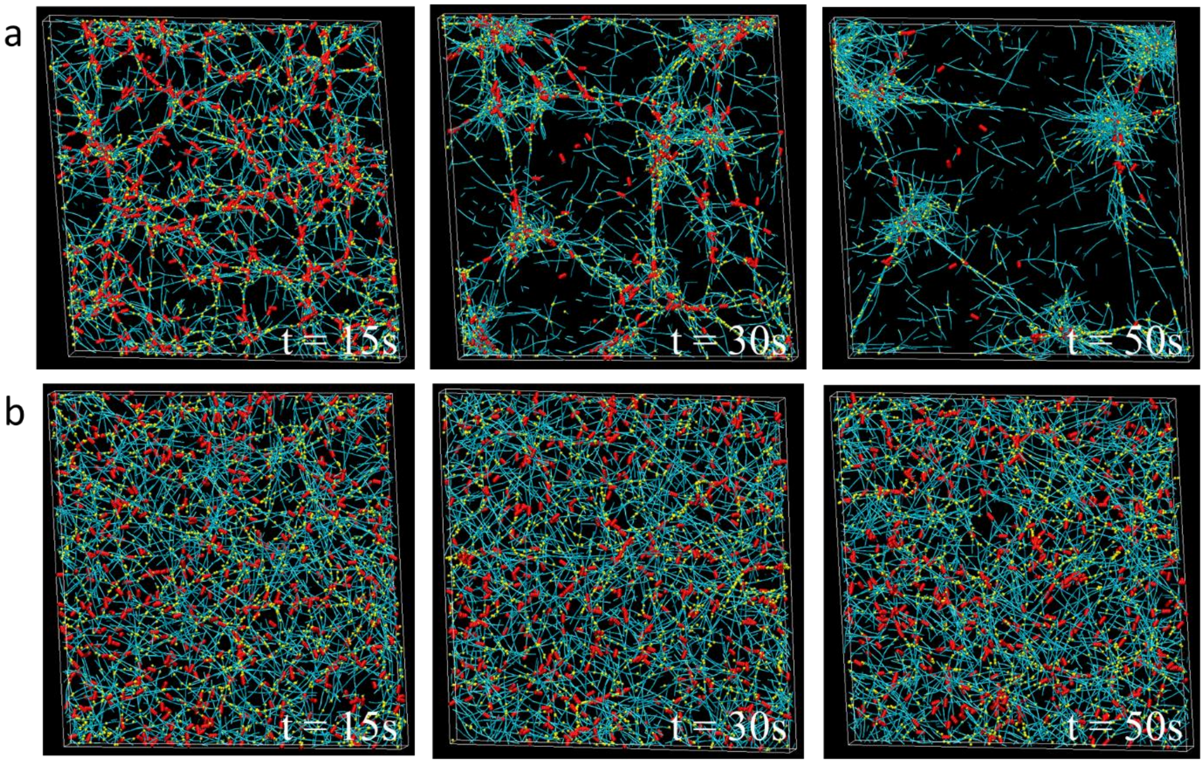

Supplementary Figure 1: Networks with the 2D cortical geometry (8 μm x 8 μm x 0.5 μm) display altered morphologies depending on the actin turnover rate. a) A 2D cytoskeletal network with an actin turnover rate of 0 s^{-1}, 25 μM actin, 1% ACPs, and 1% motors at different time points. Motors started walking at t = 10s. At t = 30s and 50s, the network has aggregated under internal stress. b) A 2D cytoskeletal network with an actin turnover rate of 200 s^{-1}, 25 μM actin, 1% ACPs, and 1% motors at different time points. Motors started walking at t = 10s. The network is homogeneous and can sustain the generated stress. Teal, yellow, and red are actin filaments, ACPs, and motors, respectively.

Supplementary Figure 2

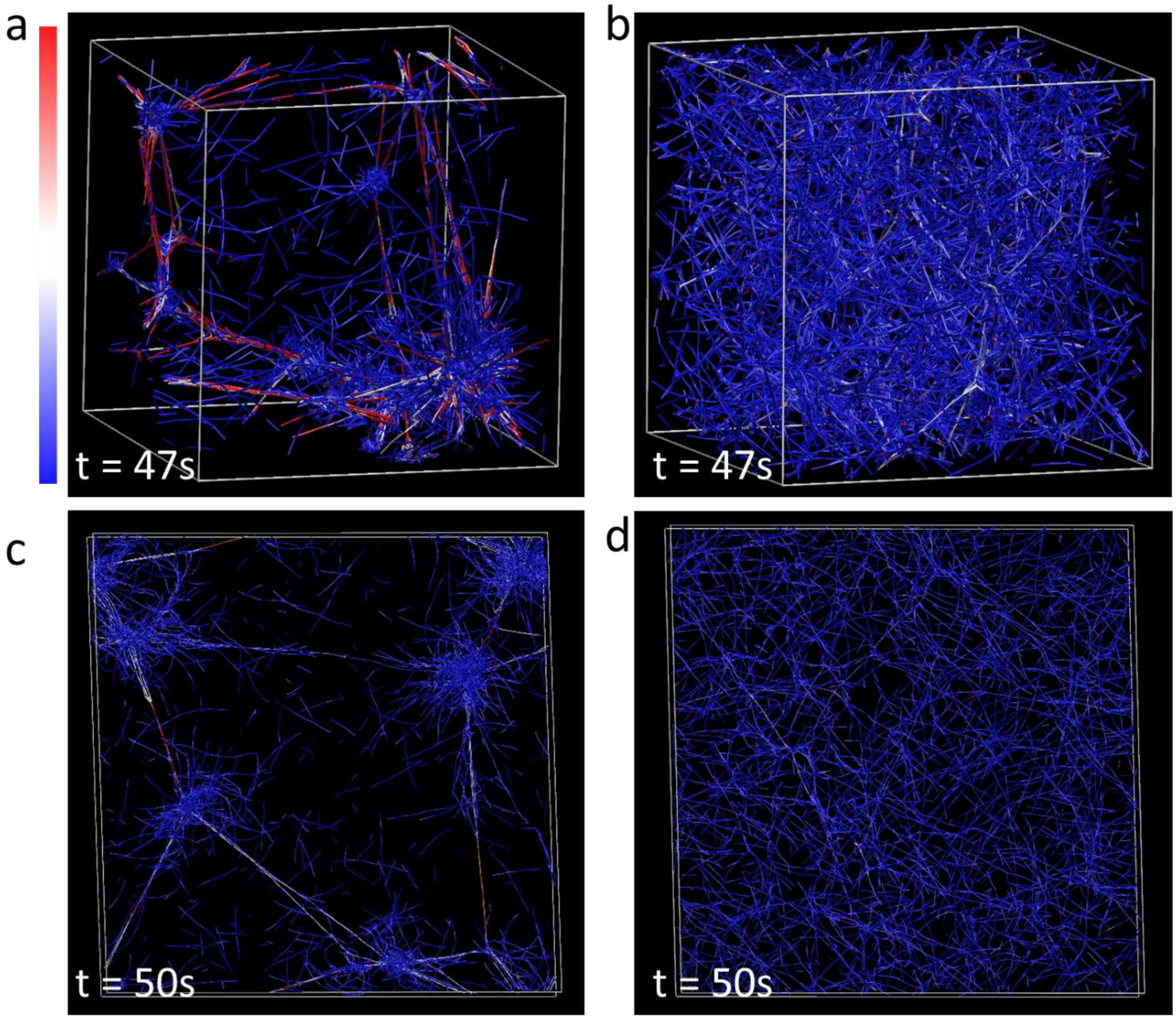

Supplementary Figure 2: Spatial distribution of internal tension. a-d) Tension profiles of networks at corresponding time points in Fig. 1a, Fig1b, Supplementary Fig. 1a, Supplementary Fig. 1b, respectively. The color bar indicates the linear scale for tension. The bottom corresponds to tensions less than or equal to 0 and the top corresponds to tensions of 100 pN or larger.

Supplementary Figure 3

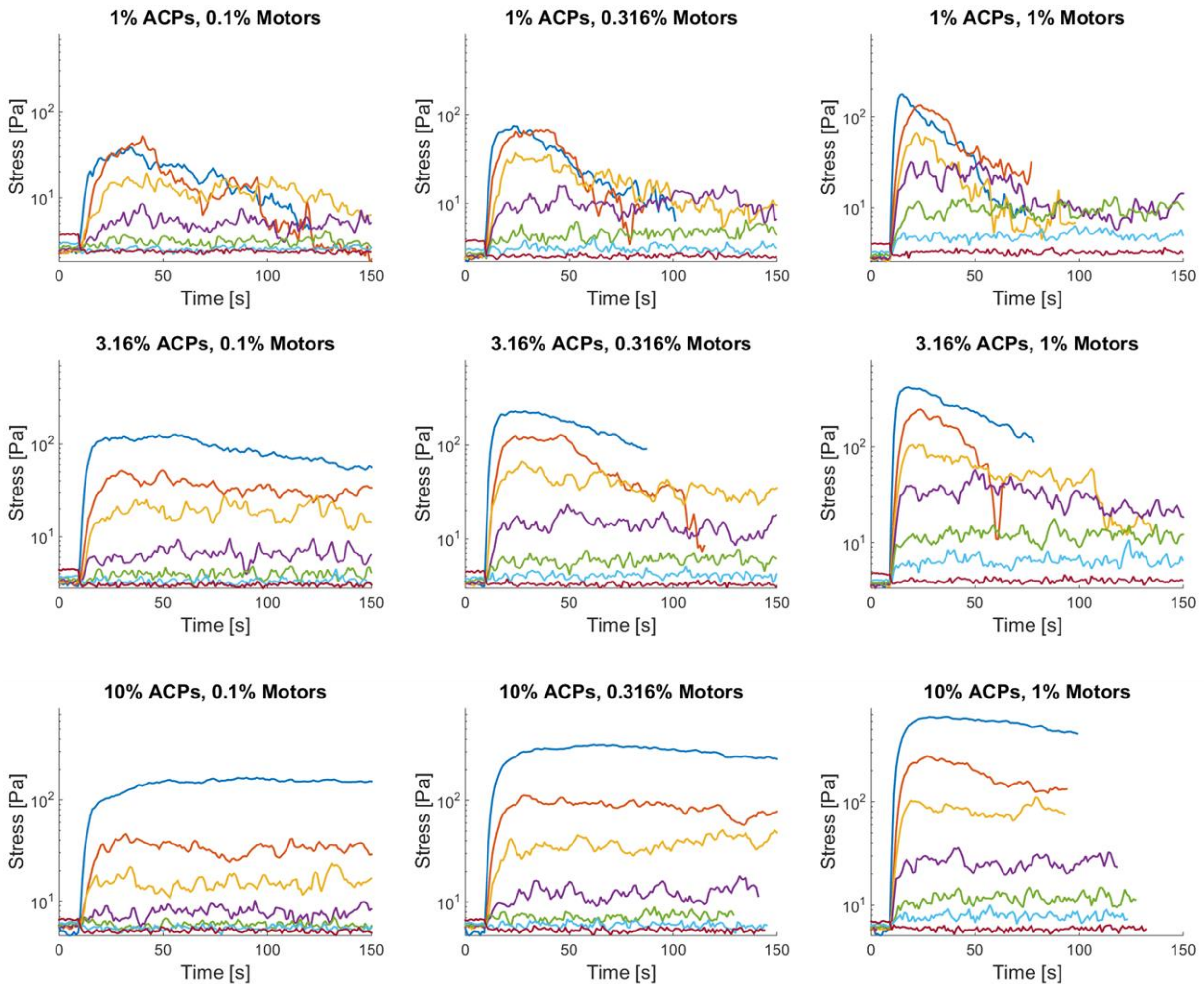

Supplementary Figure 3: Time evolution of stress profiles for 2D (8 μm x 8 μm x 0.5 μm) networks with 25 μM actin and the specified percentages of ACPs and motors to actin. Each row has the same concentration of ACPs (from 1% to 10%) and each column has the same concentration of motors (from 0.1% to 3.16%). Blue, red, yellow, purple, green, light blue, and dark red curves represent actin turnover rates of 0, 30, 60, 120, 200, 300, and 600 s^{-1}, respectively. Networks with stress profiles that peak and collapse are unstable and lead to aggregates, while networks that display sustained steady-state stress profiles (from initial motor activation) tend to stay homogeneous. The average filament length of these networks is ~1 μm.

Supplementary Figure 4

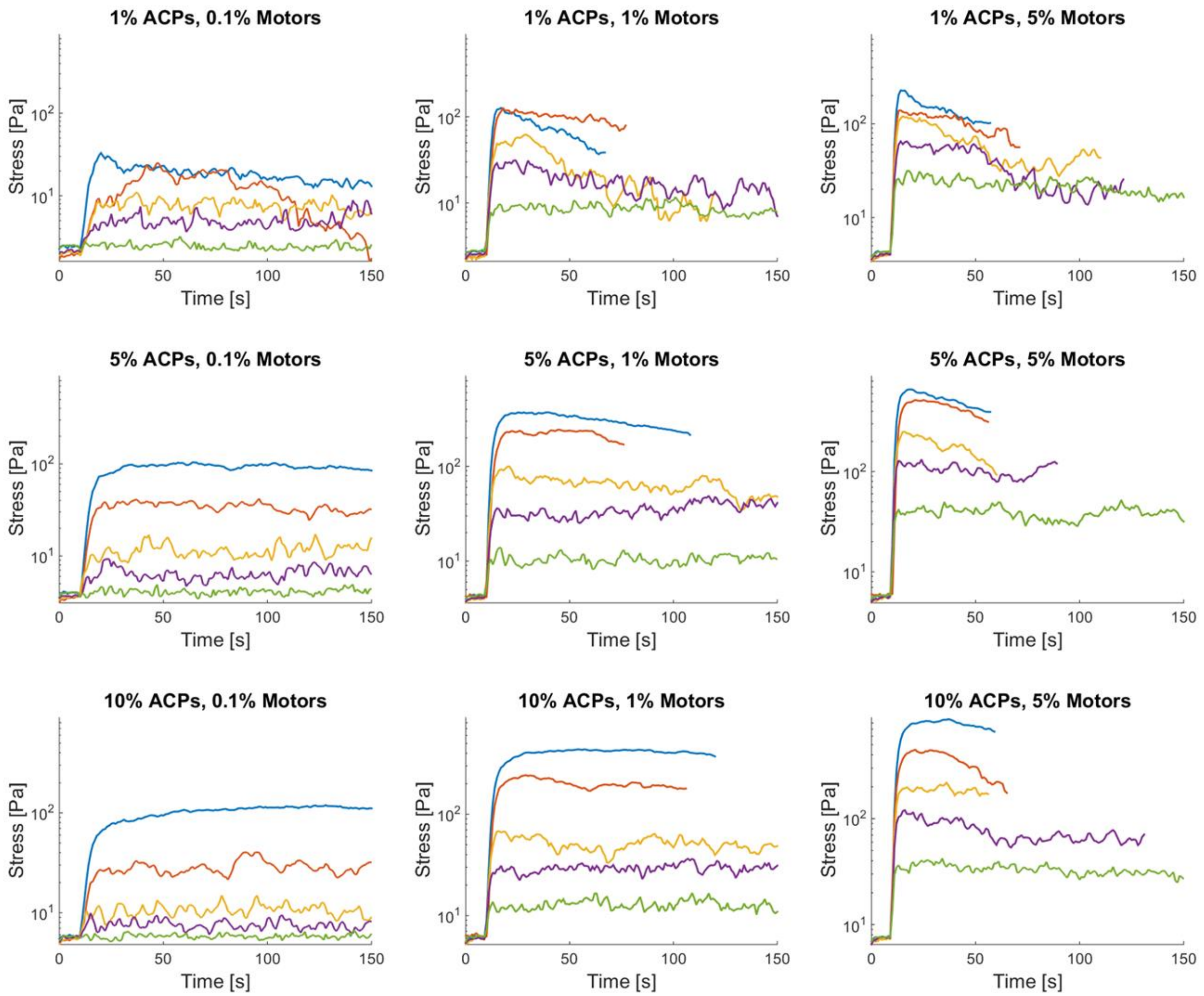

Supplementary Figure 4: Time evolution of stress profiles for various network configurations in 3D domains (3 μm x 3 μm x 3 μm) with 25 μM actin and the specified percentages of ACPs and motors to actin. Blue, red, yellow, purple, and green curves represent actin turnover rates of 0, 30, 90, 150, and 300 s^{-1}, respectively. Each column has the same motor concentration and each row has the same ACP concentration. The average filament length of these networks is ~1.3 μm.

Supplementary Figure 5

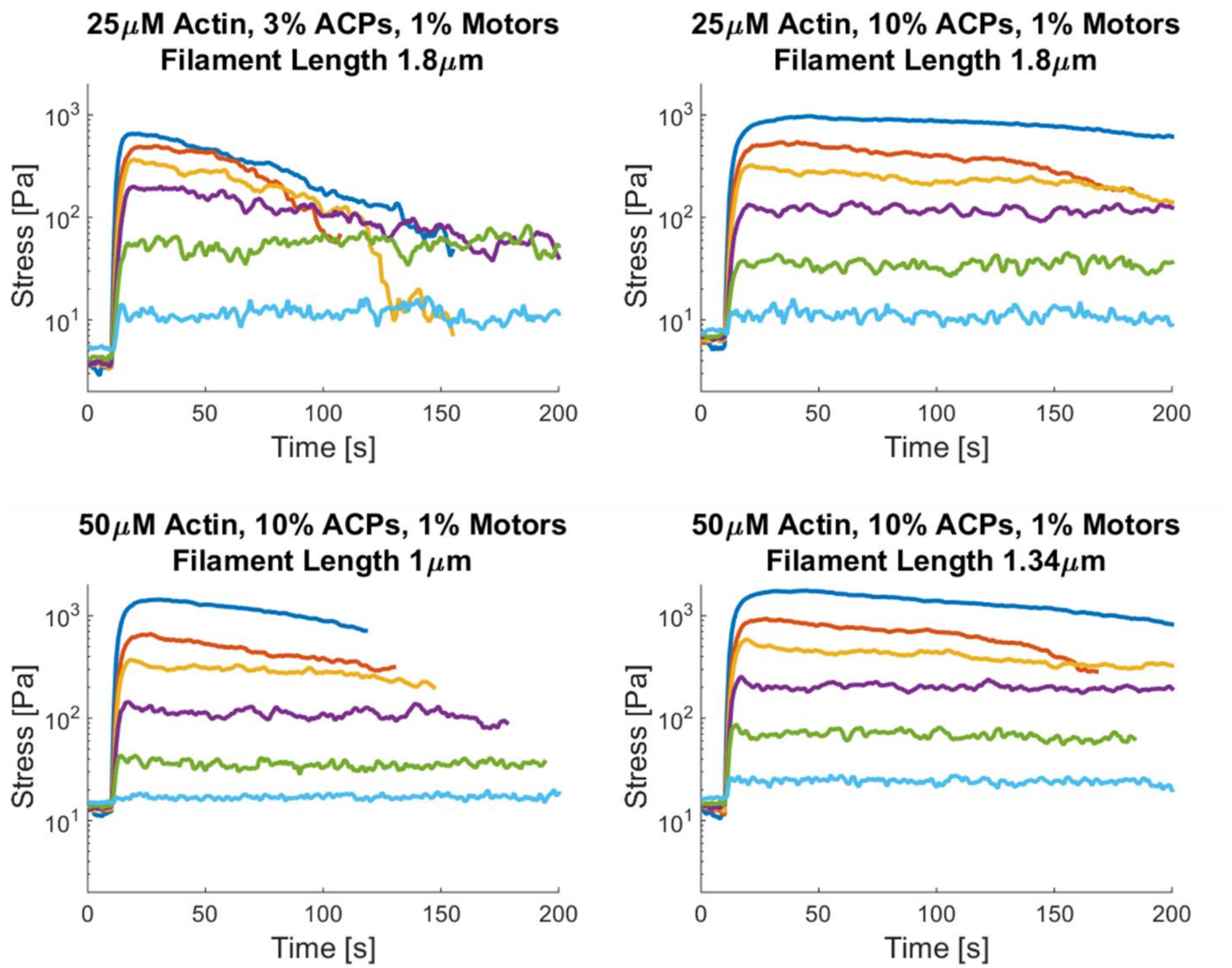

Supplementary Figure 5: Impact of concentrations and filament length on stresses measured in the 2D cortical geometry. Average filament length is varied by changing the ratio of the polymerization rate to the nucleation rate. Each curve on the same plot corresponds to a different actin turnover rate: 0, 30, 60, 120, 240, 480 s^{-1} from top to bottom, respectively. Increasing ACP concentration or actin filament length enhances the stability of the stress, especially for lower actin turnover rates, corresponding to larger stresses. Increasing filament length also leads to larger stresses. Doubling the actin concentration appears to have less of an effect (compared to Supplementary Fig. 3), but may slightly increase the stability of the network.

Supplementary Figure 6

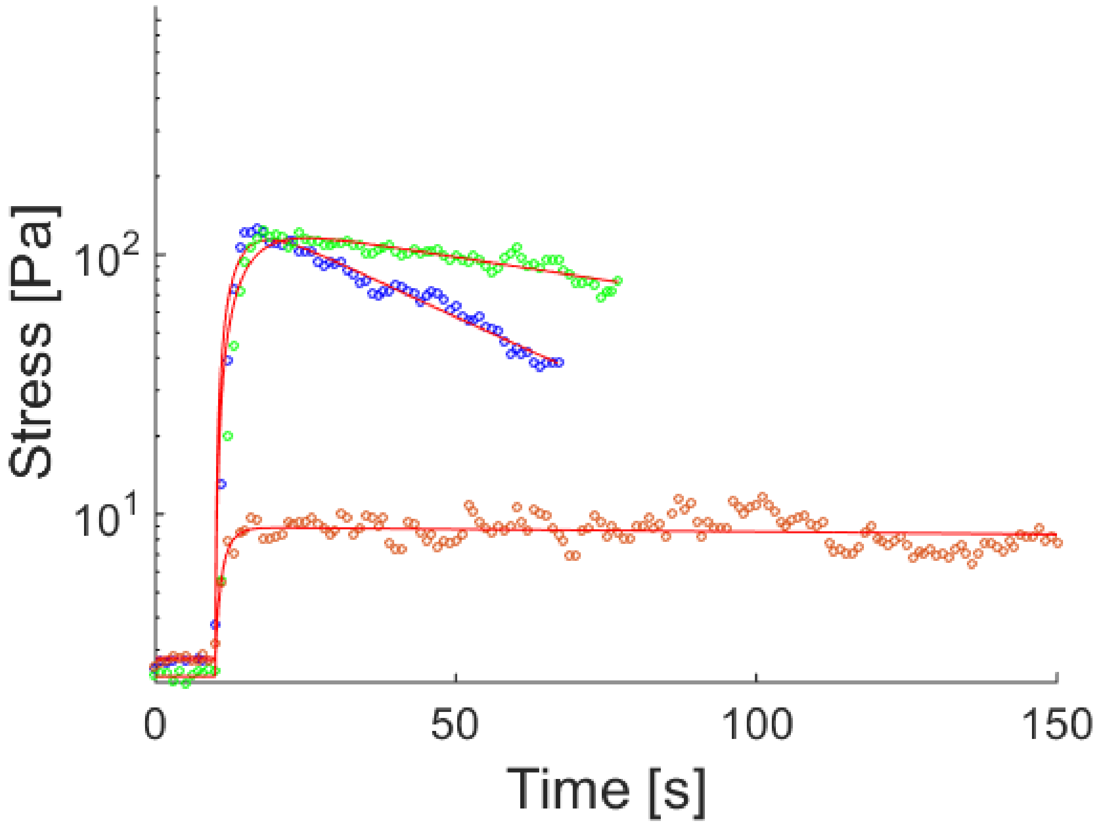

Supplementary Figure 6: Curve fits on stress profiles. Red curves are fits according to equation S2 and circles are numerical data from typical stress profiles for different simulated network configurations, including both aggregated and homogeneous networks. Data are from configurations with 25 μM actin, 1% ACPs, 1% motors, and actin turnover rates of 0 (blue), 30 (green), or 300 s^{-1} (orange).

Supplementary Figure 7

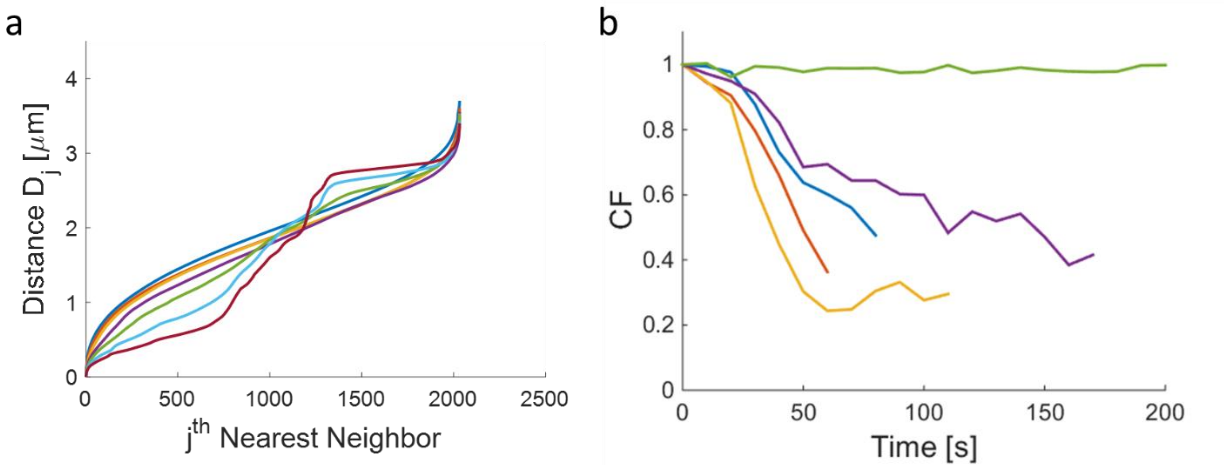

Supplementary Figure 7: Separation distances and the clustering factor. a) The separation distance D_j between neighbors evolves over time for a network that aggregates (1% ACP, 1% motors, 30 s^{-1} actin turnover rate). Dark blue, red, yellow, purple, green, light blue, and maroon curves indicate t = 0 to 60s at 10s time intervals, respectively. The signature for aggregated networks (low D_j for a large number of close neighbors) is apparent at later time points. b) The clustering factor *CF* evolves over time for a network with 1% ACP and 1% motors at different actin turnover rates. Blue, red, yellow, purple, and green curves correspond to actin turnover rates of 0, 30, 90, 150, and 300 s^{-1}, respectively. Homogeneous networks have high and stable *CF*'s whereas aggregated networks have *CF*'s that diminish over time.

Supplementary Figure 8

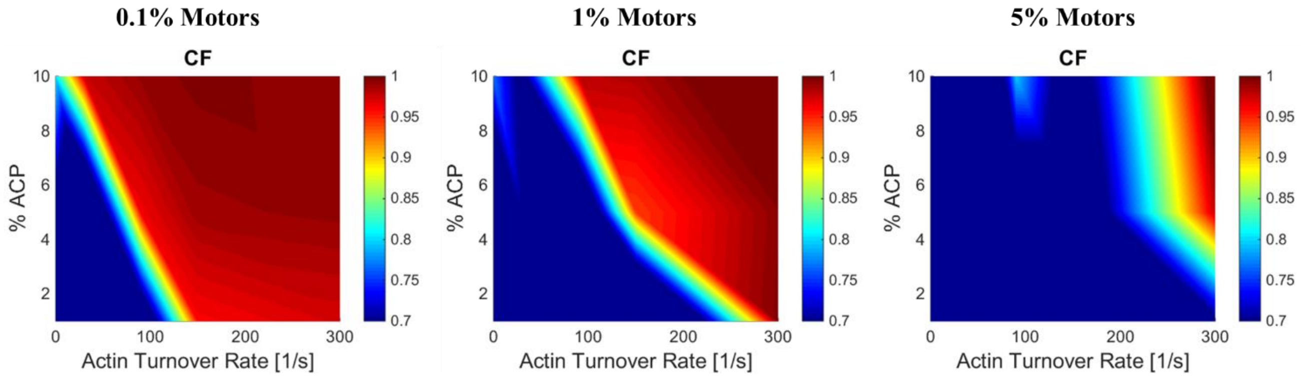

Supplementary Figure 8: Heat maps of the clustering factor (CF) measured at the end of simulation time for each configuration (see Supplementary Fig. 4 for typical durations), as described in Supplementary Note 3.

Supplementary Figure 9

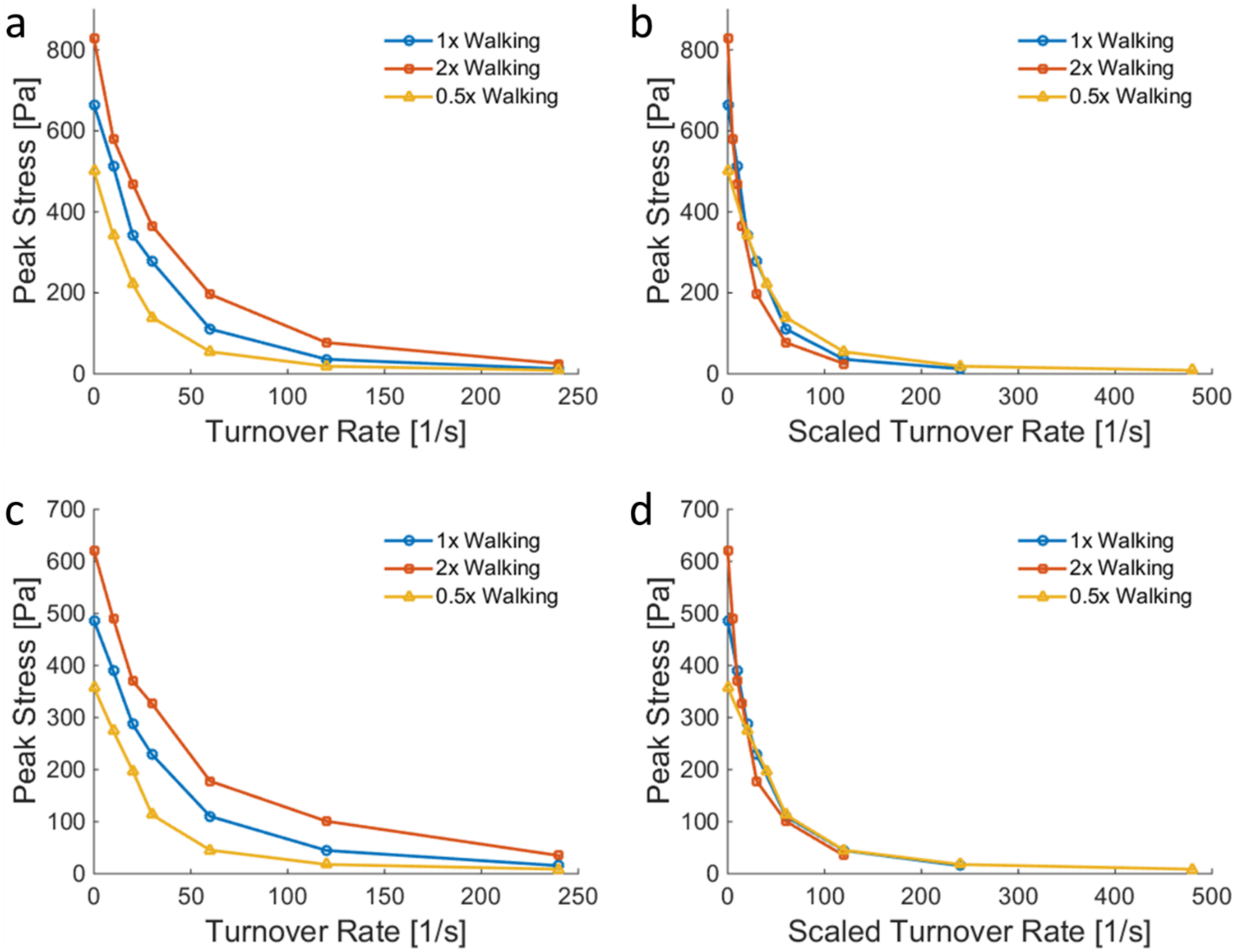

Supplementary Figure 9: Peak stresses of 2D (a,b) and 3D (c,d) networks with 25 μM actin, 10% ACPs, and 1% motors. (a,c) The peak stresses of networks at various actin turnover rates for different scaling of the motor walking rate. Increased motor walking speed increases the peak stress for the same turnover rate. (b,d) The peak stresses of networks vs. the scaled turnover rate, equal to the turnover rate divided by the scaling factor of the motor walking rate. The curves collapse, indicating that a fixed ratio of the turnover rate to motor walking rate produces the same stress profiles for relatively high (non-zero) turnover rates.

Supplementary Figure 10

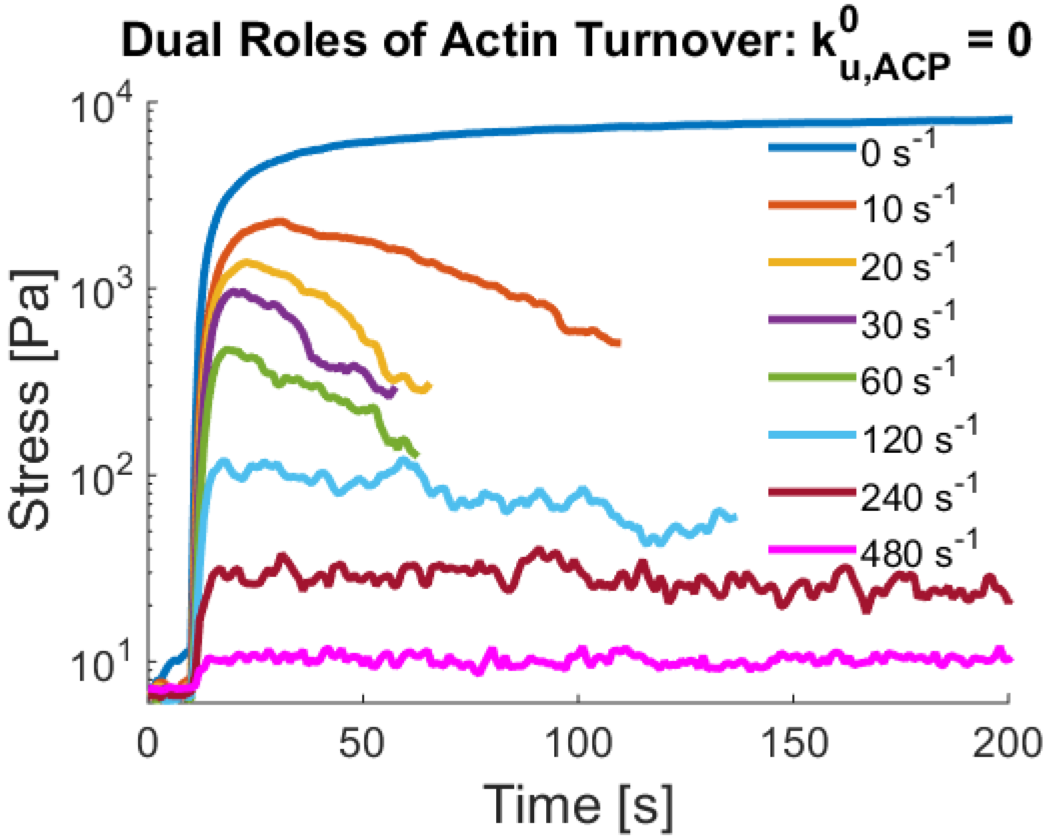

Supplementary Figure 10: Dual roles of actin turnover. With permanent ACPs ($k^0_{u,ACP} = 0$), actin turnover can serve to both diminish and sustain steady-state stresses. Numbers in the legend indicate actin turnover rates. Without any intrinsic ACP unbinding, low actin turnover rates lead to a collapse in the stress profile, while high actin turnover rates recover stable stress profiles. However, at the lowest actin turnover rate (0 s^{-1}), the stress is stable. These simulations were performed in the 2D cortical geometry (8 µm x 8 µm x 0.5 µm) with 25 µM actin, 7% ACPs, and 5% motors.

Supplementary Figure 11

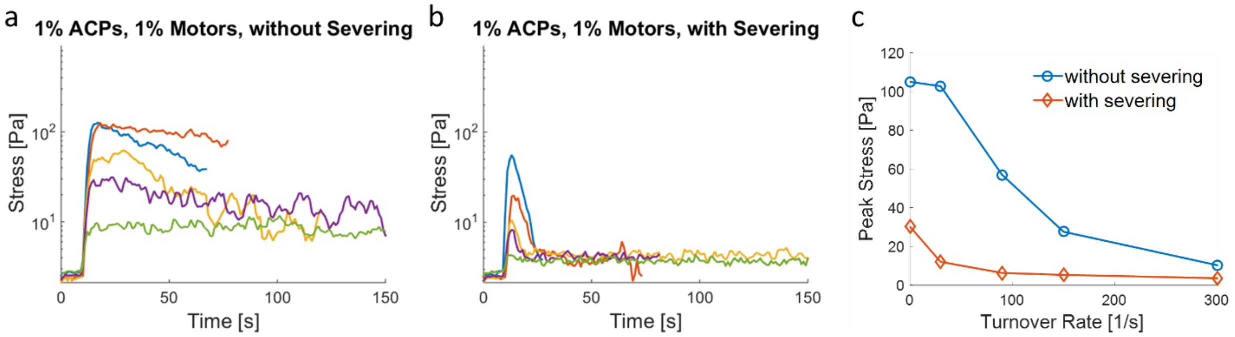

Supplementary Figure 11: Stress dynamics of 3D networks with 1% ACP, 1% motors, and 25 μM actin (a) without and (b) with bending angle-dependent severing of actin filaments. Blue, red, yellow, purple, and green curves indicate actin turnover rates (due to treadmilling) of 0, 30, 90, 150, and 300 s^{-1}, respectively. (c) Peak stress vs. turnover rate for simulations in (a) and (b). Blue circles and red diamonds represent simulations without and with severing, respectively. Peak stresses are calculated by taking the average stress over a 10 s time interval centered on the time point of maximal stress.

Supplementary Table 1: List of parameters employed in the model. Most of the parameter values are identical to those used in our recent work [1]. Parameter values without references are either determined arbitrarily due to lack of experimental data or generally accepted ones.

Symbol	Definition	Value
$r_{0,\mathrm{A}}$	Length of a cylindrical actin segment	1.4×10^{-7} [m]
$r_{\mathrm{c,A}}$	Diameter of a cylindrical actin segment	7.0×10^{-9} [m] [2]
$\theta_{0,\mathrm{A}}$	Bending angle of actin	0 [rad]
$\kappa_{\mathrm{s,A}}$	Extensional stiffness of actin	1.69×10^{-2} [N m^{-1}]
$\kappa_{\mathrm{b,A}}$	Bending stiffness of actin	2.64×10^{-19} [N m] [3]
$r_{0,\mathrm{ACP}}$	Length of an ACP arm	2.35×10^{-8} [m] [4]
$r_{\mathrm{c,ACP}}$	Diameter of an ACP arm	1.0×10^{-8} [m]
$\theta_{0,\mathrm{ACP}}$	Bending angle of ACP	0 [rad]
$\kappa_{\mathrm{s,ACP}}$	Extensional stiffness of an ACP arm	2.0×10^{-3} [N m^{-1}]
$\kappa_{\mathrm{b,ACP}}$	Bending stiffness of ACP	1.04×10^{-19} [N m]
$r_{0,\mathrm{M1}}$	Length of a bare zone of motor backbone	4.2×10^{-8} [m]
$r_{0,\mathrm{M2}}$	Length of side segments of motor backbone	4.2×10^{-8} [m]
$\theta_{0,\mathrm{M}}$	Bending angle of motor backbone	0 [rad]
$\kappa_{\mathrm{s,M1}}$	Extensional stiffness 1 of motor backbone	1.69×10^{-2} [N m^{-1}]
$\kappa_{\mathrm{s,M2}}$	Extensional stiffness 2 of motor backbone	1.69×10^{-2} [N m^{-1}]
$\kappa_{\mathrm{b,M}}$	Bending stiffness of motor backbone	5.07×10^{-18} [N m]
$r_{0,\mathrm{M3}}$	Length of a motor arm	1.35×10^{-8} [m] [5]
$r_{\mathrm{c,M}}$	Diameter of a motor arm	1.0×10^{-8} [m]
$\kappa_{\mathrm{s,M3}}$	Extensional stiffness 1 of a motor arm	2.5×10^{-4} [N m^{-1}]
$\kappa_{\mathrm{s,M4}}$	Extensional stiffness 2 of a motor arm	1.0×10^{-3} [N m^{-1}] [6]
N_{h}	Number of heads represented by a motor arm	8
N_{a}	Number of arms in a single motor	8
κ_{r}	Strength of repulsive force	1.69×10^{-3} [N m^{-1}]
C_{A}	Actin concentration	25 [μM]
% Motors	[Ratio of C_{M} to C_{A}] × 100	0.1-5
% ACPs	[Ratio of C_{ACP} to C_{A}] × 100	1-10
$\langle L_{\mathrm{f}} \rangle$	Average length of actin filaments	1.3 [μm] for 3D domain<br>1 [μm] for 2D domain
Δt	Time step	2.3×10^{-5} [s]

μ	Viscosity of medium	0.86×10^{-3} [kg m^{-1} s^{-1}]
$k^0_{\mathrm{u,ACP}}$	Zero-force unbinding rate coefficient of ACP	0.115 [s^{-1}] [7]
$\lambda_{\mathrm{u,ACP}}$	Compliance of a bond for ACP unbinding	1.04×10^{-10} [m] [7]
$k_{\mathrm{B}}T$	Thermal energy	4.142×10^{-21} [J]
$k_{a,p}$	Actin polymerization rate	30n [μM^{-1} s^{-1}]
$k_{a,d}$	Actin depolymerization rate	30n [s^{-1}]
$k_{a,n}$	Actin nucleation rate	0.01n [μM^{-1} s^{-1}]
n	Actin turnover scaling factor	0-20
k^0_{sev}	Zero-angle severing rate coefficient	1.0×10^{-17} [s^{-1}]
λ_{sev}	Sensitivity of a severing rate to an angle	2.79×10^{-2} [rad]
**For "Parallel Cluster Model"** [8,9]		
k_{01}	A rate from unbound to weakly bound state	40 [s^{-1}]
k_{10}	A rate from weakly bound to unbound state	2 [s^{-1}]
k_{12}	A rate from weakly bound to post-power-stroke state	1,000 [s^{-1}]
k_{21}	A rate from post-power-stroke to weakly bound state	1,000 [s^{-1}]
k_{20}	A rate from post-power-stroke to unbound state	20 [s^{-1}]
F_0	Constant for force dependence	5.04×10^{-12} [N]
E_{pp}	Free energy bias toward the post-power-stroke state	-60×10^{-21} [J]
E_{ext}	External energy contribution	0 [J]
d	Step size	7×10^{-9} [m]
k_{m}	Spring constant of the neck linkers	1×10^{-3} [N m^{-1}]

Supplementary Note 1: Brownian Dynamics Model of the Active Actin Cytoskeleton

We simulated actomyosin networks composed of F-actins, passive ACPs, and active myosin motors by modifying our previous model built based on Brownian dynamics [1]. Definitions and values of parameters used in the model are described in Supplementary Table 1.

Coarse-grained representations of F-actin, ACPs, and motors

Actin filaments (F-actin) are coarse-grained using cylindrical segments with polarity (barbed and pointed ends) whose length and diameter are 140 nm and 7 nm [2] respectively, serially connected by elastic hinges. ACPs are modeled as pairs of cylindrical arms of 23.5 nm in length and 10 nm in diameter serially connected to each other by elastic hinges, which mimics the geometry of α-actinin [4]. Motors consist of a relatively rigid backbone with symmetric polarity comprising multiple segments of 42 nm in length serially connected by hinges. Each endpoint of the backbone segment has two motor arms of 13.5 nm in length [5], mimicking the structure of myosin bipolar thick filaments (TF)[10]. The arms of ACPs and motors can bind to binding sites located every 7 nm on actin segments.

Brownian dynamics using the Langevin equation

Displacements of the cylindrical segments representing F-actins, ACPs, and motors are governed by the Langevin equation with inertia neglected:

$$\mathbf{F}_i - \zeta_i \frac{\mathrm{d}\mathbf{r}_i}{\mathrm{d}t} + \mathbf{F}_i^{\mathrm{T}} = 0 \qquad \text{(S1.1)}$$

where $\mathbf{r}_i$ is the location of either center point of ACPs or endpoint of segments constituting actin filaments and motor backbones, ζ_i is a drag coefficient, t is time, and $\mathbf{F}_i$ is a deterministic force that includes extensional and bending forces as well as repulsive forces. $\mathbf{F}_i^{\mathrm{T}}$, a thermal force, is determined by the fluctuation-dissipation theorem:

$$\left\langle \mathbf{F}_i^{\mathbf{T}}(t)\mathbf{F}_j^{\mathbf{T}}(t)\right\rangle = \frac{2k_{\mathbf{B}}T\zeta_i\delta_{ij}}{\Delta t}\boldsymbol{\delta} \qquad \text{(S1.2)}$$

where δ_{ij} is the Kronecker delta, $\boldsymbol{\delta}$ is a unit second-order tensor, and $\Delta t = 2.3\times10^{-5}$ s is time step. The drag coefficients of actins, ACPs, and motors are approximated as [11]:

$$\zeta_i = 3\pi\mu r_{\mathrm{c},i}\frac{3 + r_{0,i}/r_{\mathrm{c},i}}{5} \qquad \text{(S1.3)}$$

where $r_{\mathrm{c},i}$ and $r_{0,i}$ are the diameter and length of a segment, respectively. Locations of segments are updated at each time step using the Euler integration scheme:

$$\mathbf{r}_i(t+\Delta t) = \mathbf{r}_i(t) + \frac{\mathrm{d}\mathbf{r}_i}{\mathrm{d}t}\Delta t = \mathbf{r}_i(t) + \frac{1}{\zeta_i}\left(\mathbf{F}_i + \mathbf{F}_i^{\mathrm{T}}\right)\Delta t \qquad \text{(S1.4)}$$

Extensional and bending forces

Extension and bending of actins, ACPs, and motors are governed by harmonic potentials with stiffness κ_{s} and κ_{b}, respectively:

$$U_{\mathrm{s}} = \frac{1}{2}\kappa_{\mathrm{s}}(r - r_0)^2,\ U_{\mathrm{b}} = \frac{1}{2}\kappa_{\mathrm{b}}\left(\theta - \theta_0\right)^2 \qquad \text{(S1.5)}$$

where r is a distance, θ is a bending angle, and the subscript 0 denotes the equilibrium value.

i) Actin: Extensional ($\kappa_{s,A}$) and bending stiffnesses of F-actins ($\kappa_{b,A}$) maintain an equilibrium length of actin segments ($r_{0,A}$= 140 nm) and an equilibrium angle formed by adjacent actin segments ($\theta_{0,A}$ = 0 rad). We chose the value of $\kappa_{b,A}$ leading to the persistence length of 9 μm [3], but employed a lower value for $\kappa_{s,A}$ than experimental measurement [12] for a resonable computational efficiency.

ii) ACP: Extensional ($\kappa_{s,ACP}$) and bending stiffnesses of ACPs ($\kappa_{b,ACP}$) keep an equilibrium length of ACP arms ($r_{0,ACP}$ = 23.5 nm) and an equilibrium angle formed by two ACP arms ($\theta_{0,ACP}$ = 0 rad), and their values are set to reasonable values due to lack of experimental data as in our previous works [13].

iii) Motor: Extensional stiffness of motor backbone ($\kappa_{s,M1}$ and $\kappa_{s,M2}$) maintains an equilibrium length of the backbone ($r_{s,M1}$=$r_{s,M2}$= 42 nm), and their values are identical to $\kappa_{s,A}$, whereas its bending stiffness ($\kappa_{b,M}$) keeping the backbone straight ($\theta_{0,M}$ = 0 rad) is much larger than $\kappa_{b,A}$ because motors need to be capable of extending and bending actin filaments without significant deformations on themselves. Extension of each motor arm is governed by a two-spring model where a transverse spring ($\kappa_{s,M3}$) maintains an equilibrium distance ($r_{0,M3}$= 13.5 nm) between F-actin and the endpoint of the motor backbone while a longitudinal spring ($\kappa_{s,M4}$) helps maintaining a right angle formed by axis of the F-actin and a motor arm ($r_{0,M4}$= 0 nm). The value of $\kappa_{s,M4}$ is set based on experimental measurement [6].

Repulsive forces

Repulsive forces due to volume-exclusion effects are computed via a harmonic potential [13]:

$$U_{\mathrm{r}} = \begin{cases} \frac{1}{2}\kappa_{\mathrm{r}}\left(r_{12} - r_{\mathrm{c,A}}\right)^2 & \text{if} \quad r_{12} < r_{\mathrm{c,A}} \\ 0 & \text{if} \quad r_{12} \geq r_{\mathrm{c,A}} \end{cases} \tag{S1.6}$$

where r_{12} is a minimum distance between a pair of actin segments, and κ_{r} is strength of repulsive force.

Dynamics of ACPs

ACPs transiently cross-link pairs of F-actins without preference for angle of contact. ACPs unbind from F-actins in a force-dependent manner:

$$k_{\mathrm{u,ACP}} = \begin{cases} k_{\mathrm{u,ACP}}^{0} \exp\left(\dfrac{\lambda_{\mathrm{u,ACP}}\left|\vec{F}_{\mathrm{s,ACP}}\right|}{k_{\mathrm{B}}T}\right) & \text{if } r \geq r_{0,\mathrm{ACP}} \\ k_{\mathrm{u,ACP}}^{0} & \text{if } r < r_{0,\mathrm{ACP}} \end{cases} \tag{S1.7}$$

where $\vec{F}_{\mathrm{s,ACP}}$ is an extensional force acting on an ACP arm, $k_{\mathrm{u,ACP}}^{0}$ is the zero-force unbinding rate coefficient, $\lambda_{\mathrm{u,ACP}}$ is the force sensitivity of unbinding, and $k_{\mathrm{B}}T$ is thermal energy. The reference values of $k_{\mathrm{u,ACP}}^{0}$ ($k_{\mathrm{u,ACP}}^{0*}$ = 0.115 s^{-1}) and $\lambda_{\mathrm{u,ACP}}$ (= 1.04×10^{-10} m) are determined based on a previous single-molecule experiment [7], corresponding to those of filamin A.

Dynamics of motors

Bipolar TF structure of motors is formed via nucleation of a bare zone followed by symmetric assembly of backbone segments at both sides of the bare zone, leading to TFs with

constant backbone length. Each of N_a motor arms attached to a single backbone represents N_h myosin heads. A free arm can bind to a binding site located on F-actin at a rate, $40N_h$ s^{-1}. Motor arms walk on and unbind from F-actins. At each walking event, arms slide from a current binding site to a next one located toward the barbed end by ~7 nm. After reaching the barbed end, motors slide off from F-actin at a next walking event. The walking ($k_{w,M}$) and unbinding rates ($k_{u,M}$) vary depending on forces exerted on the arms and determined by "parallel cluster model" (PCM) for mechanochemical rates, step size, and the number of heads [8,9]. Table S1 shows values for the major parameters that we used for adopting PCM to our model. We assume that only forces acting on the longitudinal spring ($\vec{F}_{s,M4} = \nabla U_{s,M4}$) affect $k_{w,M}$ and $k_{u,M}$. Note that we assume that myosin heads behave as a catch bond [14,15], leading to lower $k_{w,M}$ and $k_{u,M}$ with larger applied forces, which is opposite to the tendency of ACPs. Mechanochemical rates that we used for PCM result in unloaded walking velocity of ~140 nm/s and stall force of f_M^{stall} ~5.7 pN. In our recent work [16], it was demonstrated that arms attached to a single backbone are mechanically coupled; the motor with discrete N_a arms representing N_h myosin heads per each exhibits the force-velocity relationship and dwell time corresponding to those of a myosin TF with N_aN_h heads predicted by PCM, regardless of extent of coarse-graining (N_h). Both N_h and N_a are set to 8, corresponding to 64 myosin heads per TF which is comparable to the experimentally determined size of non-muscle myosin TFs [17].

Severing of F-actin

To test effects of F-actin severing on contractile behaviors, we modeled the severing

event as disappearance of a single actin segment on a filament. A severing rate is determined by the sum of two bending angles formed by a segment and its adjacent ones according to the following equation:

$$k_{\mathrm{sev}} = k_{\mathrm{sev}}^{0} \exp\left(\frac{\theta}{\lambda_{\mathrm{sev}}}\right) \qquad \text{(S1.8)}$$

where k_{sev}^{0} is a zero-angle severing rate coefficient, and λ_{sev} is a sensitivity to the angle. Equation S1.8 and the values of k_{sev}^{0} and λ_{sev} were found in an empirical fashion; distribution of angles at which severing spontaneously occur due to thermal fluctuation in the simulations was calculated and compared with the experimental observation (57 ± 9°) [18].

Actin turnover rates

Within cells, a multitude of actin binding proteins and nucleators including Arp2/3, formins, cofilin, and myosin and relevant signaling pathways regulate actin turnover. Due to its complexity, it is hard to estimate the turnover rate at any transient moment during development, morphogenesis, and disease progression. Therefore, we explore a range of rates that is physiologically plausible and consistent with prior experimental studies. Diffusion limits spontaneous actin polymerization to be around 10 μM^{-1} s^{-1} for ATP-actin binding to the barbed end [19,20]. However, the formin mDia1 in the presence of profilin has been demonstrated to increase the rate constant of barbed end polymerization to over 100 μM^{-1} s^{-1} [20]. Furthermore, spontaneous actin depolymerization happens in a diffusion limited rate of around ~0.2 s^{-1} [21], but actin regulatory proteins such as ADF/cofilin have been shown to help significantly increase the breakdown of actin filaments via enhanced depolymerization [22,23] or severing (at low

concentrations of cofilin) [24,25]. As a result, *in vivo* turnover rates of actin filaments, as measured in studies of actin-based motility in cells and bacteria, can be over 100-fold [21] larger than spontaneous actin turnover rates.

Among many modes of actin turnovers, we primarily consider actin treadmilling for simplicity. In the model, actin filaments can polymerize, depolymerize, and nucleate from monomers with tuned rates. In each simulation, we imposed identical values for the polymerization and depolymerization rate constants, which lead to a dynamic equilibrium at C_A = 1 μM. We also maintained a fixed ratio of depolymerization to nucleation rates in order to obtain a consistent actin filament length (1 μm on average for 2D domains and 1.3 μm for 3D domains). Typical values of actin filament length measured inside cells are on the order of hundreds of nanometers to microns [26-28], and studies of reconstituted actin and actomyosin networks typically use filament lengths on the order of several microns [29,30]. We chose a range of polymerization rates from 0 to 300 $\mu M^{-1}s^{-1}$ and a range of depolymerization rates from 0 to 300 s^{-1}, which should span physiologically relevant values.

Network formation

As in our previous study [1], cross-linked actomyosin networks are assembled via self-assembly of actin monomers (G-actin), ACPs, and motors within a three-dimensional rectangular domain (3×3×3 μm for 3D networks and 8×8×0.5 μm for 2D networks) with periodic boundary condition (PBC) except in z direction of a domain for 2D networks. During the self-assembly, G-actin is polymerized into F-actin, and the TF structure of motors is formed via nucleation and polymerization of motor backbone segments with their arms binding to F-actin without walking events. ACPs also bind to F-actin, forming functional cross-links between pairs of actin

filaments. The result is an initial network that is homogeneous prior to motor walking activity under various actin turnover rates.

Supplementary Note 2: Calculation of Stress and Sustainability

For the calculation of stress generated by networks, we consider 8 cross-sections that are regularly located in the computational domain in each direction. (The z direction is not considered for the 2D domain.) Stress is calculated by summing the normal component of extensional forces of all constituents crossing a cross-section and then dividing by the area of the cross-section. We repeat this calculation on multiple cross-sections (16 for 2D and 24 for 3D) and compute the average.

In order to calculate the sustainability of steady stresses generated by the actomyosin network, we fit the stress data to a three parameter function:

$$Stress = H(t - t_0) * e^{-b*(t-t_0)} * a * \left(1 - e^{-c*(t-t_0)}\right) + d \qquad \text{(S2)}$$

where H is the Heaviside function centered at t_0, t_0 is the time the motors start walking, a is a scaling factor, c is the initial stress build up rate following motor activation, d is the background stress (from thermal motion and ACP binding/unbinding) prior to motor activation which serves as an offset, and b is the decay rate of internal stresses. Since we are interested in whether stresses can be sustained in a given cytoskeletal network, b should be related to the sustainability factor. Moreover, we normalize b to the maximum b found in all the simulations in Fig. 3 in order to provide a relative metric between different networks. We define the sustainability of the networks S to be $1 - b/b_{max}$. Therefore, the more stable a network is, the closer S is to 1 and

the more unstable the network is, the closer S is to 0. As shown in Supplementary Fig. 6, our computational data appear to fit very well to equation S2.

Supplementary Note 3: Quantification of Clustering Dynamics

Actomyosin networks cluster under certain conditions, so here we describe a metric to quantify the clustering dynamics in our simulations. We consider the positions of each ACP bound to actins in the network. We calculate the separation distance d_{ij} between the ith ACP and the jth ACP (i and j are indices for keeping track of individual ACPs):

$$d_{ij} = |\boldsymbol{r_i} - \boldsymbol{r_j}| \qquad \text{(S3.1)}$$

where $\boldsymbol{r_i}$ is the position vector of the ith ACP. We then sort d_{ij} along j from nearest to farthest for each i and average over all i to obtain the average distance D_j between an ACP and its jth nearest neighbor:

$$D_j = \frac{\sum_{i=1}^{n} sort(d_{ij}, j)}{n} \qquad \text{(S3.2)}$$

where n is the total number of bound ACPs. Note that increasing ACP concentration decreases the distance between nearest neighbors. Thus to obtain comparable results between different networks, we under-sample each configuration by C_{ACP}/1μM, *i.e.* we consider only one out of every C_{ACP}/1μM particles in our analysis, where C_{ACP} is the ACP concentration of the network.

This effectively normalizes the initial ACP separation distances to those of the 1μM configuration. We then compute $D_j(t)$ at different times t after motor activation, as demonstrated in Supplementary Fig. 7a.

We define the clustering factor CF to be the average distance between an ACP and its N nearest neighbors, normalized by that of the initial network state before significant motor activity (CF_0):

$$CF = \frac{1}{CF_0} * \frac{\sum_{j=1}^{N} D_j}{N} \qquad \text{(S3.3)}$$

where N is chosen to be 500 (out of ~2000). Clustered networks will have a low CF, whereas sparse networks will have a high CF ~ 1. CF can be calculated for each time point simulated (Supplementary Fig. 7b).

To compare between network states, we calculated CF at a fixed time point after motor activation, t = 40s, and plot the heat maps of CF for various % ACPs, % motors, and actin turnover rates (Fig. 3c). We further check whether networks are severely clustered by the end of their simulation time $CF(t_f)$ (Supplementary Fig. 8). Severe clustering is defined to be $CF < 0.7$, which visually appears to be highly clustered. Therefore, we threshold the lowest color on CF heat maps to be 0.7, *i.e.* $CF < 0.7$ shows as blue on the heat maps. Note that simulations that end earlier due to computational cost are typically severely clustered. Networks that are still homogeneous after the end of their simulation time are typically steady and can sustain homogeneity for over 150 s.

Supplementary Note 4: Altered Binding and Turnover Dynamics

Effect of ACP Unbinding Rate

We investigated the interplay between actin turnover and ACP unbinding to determine their relative roles in network stress profiles and morphologies. We reduced the unbinding rate of ACPs to 0, which means that the ACPs do not unbind intrinsically and only unbind when actin filaments depolymerize at the binding sites. As shown in Supplementary Fig. 10, without any actin turnover, the internal stress of the network builds up to much larger levels than in previous cases and is sustained. However, at low actin turnover rates, network aggregation reappears. As actin filaments are turned over, ACPs unbind from disappearing filaments and the stress that they held is dissipated, leading to aggregation. At high actin turnover rates, however, the network can sustain steady-state stresses, as before.

Physiologically, different ACPs have different unbinding rates. For example, fascin [31] and scruin [32] are relatively permanent. Furthermore, different isoforms of ACPs and some adhesion proteins may possess different bond behavior, such as catch bonds that have decreased unbinding rates under tension [33]. Our results show that with ACPs that have more permanent bond characteristics, actin turnover can serve dual, opposing functions of both destabilizing and stabilizing stress profiles.

Impact of Actin Severing

The bending of actin filaments facilitates fragmentation [18,34]. Past a critical bending angle, actin filaments are more likely to sever, thus promoting turnover and stress relaxation. Myosin motors can enhance filament bending and breaking [34] and contribute to cell-scaled actin

network treadmilling [35]. We investigate this effect here by introducing a bending angle-dependent severing rate in our simulations (see Supplementary Note 1 for details). Our results show that the incorporation of severing activity leads to lower stresses and faster stress decay (Supplementary Fig. 11). This further demonstrates the interdependence of cytoskeletal components in regulating global network properties. Future investigations incorporating other physiological cytoskeletal features, such as additional potential mechanisms of myosin induced actin turnover, *e.g.* disassembling actin bundles and enhancing depolymerization [36] and selectively disassembling anti-parallel actin structures [37], and other actin dynamics modifiers, *e.g.* capping and branching, are necessary to elucidate detailed *in vivo* dynamics.

Supplementary Note 5: Modulating Active Stress Fluctuations

Molecular motors, in addition to generating intracellular prestress and transporting cargo in a directed manner, also provide increased fluctuations throughout the cytoskeletal network, as demonstrated by the enhanced, random but non-thermal motions seen in intracellular organelles and exogenously injected nanoparticles [38-42]. The true biological function of this added noise is not yet clear, but transport enhancement can play critical roles in redistributing macromolecules and metabolic factors, particularly in crowded and compartmentalized networks such as the intracellular space [43-46]. Additionally the intracellular environment is mechanosensitive, where signals can be activated by force. For example, ACPs such as filamin have cryptic binding sites that become accessible when they are stretched [47]. Motor induced stress fluctuations can provide a means to dynamically modulate mechanotransduction in time and space, enabling an added dimension in signal encoding.

Our simulation results demonstrate that actin turnover dynamics and motor concentration can regulate the magnitude of stress fluctuations. As shown in Fig. 5, as the actin turnover rate decreases, the distribution of changes in stress over time, taken to be the change in stress over 1 second time intervals, widened, indicating that that magnitude of stress fluctuations is enhanced. Furthermore, at the same actin turnover rate, increased motor density can lead to enhanced stress fluctuations. This enhancement can lead to a larger dispersion of intracellular macromolecules and modulate stress-dependent reaction cascades. Keeping the same motor concentration and actin turnover rate while modulating ACP concentrations does not appear to alter the stress fluctuation distribution, at least in the regime with high actin turnover relative to intrinsic ACP unbinding. Fig. 5d shows the amplitude of stress fluctuations in relation to motor and ACP

concentrations and actin turnover. These results suggest that active factors – actin (de)polymerization and motor force generation – are key determinants of enhanced stress fluctuations in cytoskeletal networks. Activating motors through VEGF (vascular endothelial growth factor) stimulation of ROCK (Rho-kinase), which regulates the phosphorylation of myosin, has been shown via particle tracking microrheology experiments to enhance intracellular fluctuations of endothelial cells [48]. Inhibition of myosin IIa via blebbistatin suppresses active fluctuations [38]. Further experiments are required to investigate how precisely tuning actin turnover impacts intracellular fluctuations. Because our simulations entail the dynamics and interactions of individual elements, we are able to recover "biological noise" (stress fluctuations) that may otherwise be overlooked in continuum approaches.

Supplementary Note 6: Active Kinetic Spring Model

Based on our *in silico* results and in order to better understand conceptually complex intracellular mechanics, we derived an analytical model that captures the dynamics of motorized cytoskeletal networks. We considered the network as a primarily elastic material with a spring constant that is dependent on time and the forces acting on it.

$$F = K(t) * x \tag{S6.1}$$

where F is the total internal force in the network (analogous to the stress), K is the dynamic spring constant (the modulus) dependent on time t, and x is the effective stretch of the network from equilibrium (the strain of the actin network internally by motors). The dynamics of this spring depends on the unbinding of force-sensitive ACPs, which reduces the spring constant, and the turnover and reformation of new crosslinked filaments, which helps the spring constant recover, as described by:

$$\frac{dC_\mathrm{b}}{dt} = k_\mathrm{b} C_\mathrm{u} - k_\mathrm{u} C_\mathrm{b} \tag{S6.2}$$

where C_b is the number of bound ACPs that help maintain the integrity of the spring, C_u is the number of ACPs that are unbound, k_b is the rate of binding or reforming of the crosslinks, and k_u is the unbinding rate of bound crosslinks. The total number of crosslinks is assumed to be conserved so $C_\mathrm{tot} = C_\mathrm{b} + C_\mathrm{u}$. Additionally, the spring constant is directly proportional to the concentration of bound crosslinks, $K = q * C_\mathrm{b}$, where q is a scaling constant. In the simple case of all springs in parallel as shown in Fig. 6a, q is the spring constant of each ACP-actin spring construct. For simplicity, we assume that the rates are constant or have quickly reached a steady level in time. Then, the solution to equation S6.2 is:

$$C_{\mathrm{b}}(t) = \frac{C_{tot}}{k_{\mathrm{b}}+k_{\mathrm{u}}}\left(k_{\mathrm{b}} + k_{\mathrm{u}}\mathrm{e}^{-(\mathrm{k_b}+\mathrm{k_u})t}\right) \tag{S6.3}$$

assuming that at t = 0, all ACPs responsible for network elasticity are in the bound state and the network has its maximum spring constant before remodeling by motors.

k_{b} and k_{u} are dependent on several important parameters. k_{b} is proportional to the F-actin concentration in the network, C_{A}, since ACPs have to bind to F-actin to form functional crosslinks. In addition, it is assumed that the average amount of force acting on each ACP affects k_{b} because it requires more work to reform a crosslink that was under high tension. Based on the Arrhenius equation, k_{b} is proportional to $\exp(-E/k_{\mathrm{B}}T)$, where E is the sum of activation energy and mechanical work required for binding or rebinding. The mechanical component of the binding rate is then:

$$k_{\mathrm{b}} \propto \exp\left(-\lambda_{\mathrm{b}}\frac{\langle F_{\mathrm{ACP}}\rangle}{k_{\mathrm{B}}T}\right) \tag{S6.4}$$

where $\langle F_{\mathrm{ACP}}\rangle$ is the average force acting on each ACP and λ_{b} is related to the recoil distance of the spring constructs after ACP unbinding. $\langle F_{\mathrm{ACP}}\rangle$ and λ_{b} should both be roughly proportional to the ratio of the concentration of motors, C_{M}, to C_{tot} because motors generate forces and strain the network, whereas ACPs support the motor-induced forces and distribute strains. Therefore, the effective binding rate is:

$$k_{\mathrm{b}} = k_{\mathrm{b0}}C_{\mathrm{A}}\exp\left(-b\left(\frac{C_{\mathrm{M}}}{C_{\mathrm{tot}}}\right)^2\right) \tag{S6.5}$$

where k_{b0} is the zero-force binding rate, and b is a scaling constant.

k_{u} also depends on force according to Bell's equation [49]:

$$k_{\mathrm{u}} = k_{\mathrm{u0}}\exp\left(\frac{\lambda_{\mathrm{u}}\langle F_{\mathrm{ACP}}\rangle}{k_{\mathrm{B}}T}\right) = k_{\mathrm{u0}}\exp\left(a\frac{C_{\mathrm{M}}}{C_{\mathrm{tot}}}\right) \tag{S6.6}$$

where k_{u0} is the zero-force unbinding rate, λ_u is the mechanical compliance of the ACP, and a is a scaling constant. Since not all ACPs bear stress in a disordered network (as simulated in our Brownian dynamics model), k_{u0} in the active kinetic spring model should be a fraction of the actual zero force unbinding rate of an ACP. Here, we assume this fraction to be 1/3 so k_{u0} is 0.038 s^{-1}. We further make an assumption that k_{b0} is 0.038 $\mu M^{-1}\ s^{-1}$, so the intrinsic binding rate is much higher than the unbinding rate at zero-force for 25 μM actin and most ACPs are bound in steady-state. Note that we assumed that k_b and k_u are independent of time when we analytically solved equation S6.1. This assumption was based on the approximation that each spring construct in the network experiences a constant tensile force (the average force). For simplicity and qualitative evaluation, we assume that a and b are 1. The chosen value for b leads to a substantial decay of k_b when $\frac{C_\mathrm{M}}{C_\mathrm{tot}}$ is 1, which corresponds to results obtained in our Brownian dynamics simulation results in which decaying stress profiles emerge when $\frac{C_M}{C_{tot}}$ is on that order.

k_b and k_u also depend on the turnover rate of the actin filaments, since turnover dynamics helps regenerate the network by causing the unbinding of motors and ACPs as well as inducing new stress-bearing bonds to form. Therefore, the total binding and unbinding rates need to reflect the turnover rates:

$$k_\mathrm{u} = k_\mathrm{u0} \exp\left(a \frac{C_\mathrm{M}}{C_\mathrm{tot}}\right) + k_\mathrm{d} \qquad \text{(S6.7)}$$

$$k_\mathrm{b} = C_\mathrm{A} \left(k_\mathrm{b0} \exp\left(-b \frac{C_\mathrm{M}}{C_\mathrm{tot}}\right) + r k_\mathrm{p}\right) \qquad \text{(S6.8)}$$

where r is the ratio of monomeric to filamentous actin, which is around 0.035 based on our Brownian dynamics simulations for the investigated configurations. k_p and k_d are the actin

polymerization and depolymerization rates, respectively. At steady-state actin turnover, which we assume, $k_{\mathrm{d}} = C_{\mathrm{A}} r k_{\mathrm{p}}$.

The effective deformation of the network from equilibrium, x in equation S6.1, is assumed to be the average of the motor-induced extensions of spring constructs that cross the cross-section of a domain. For simplicity, we assume that motors walk at a constant speed. This leads to a constant network deformation rate up to a certain characteristic time t_1 until further deformation stalls, which results from a dynamic steady-state caused by a balance between motors actively walking and motors being stalled, motors walking off of filament ends, filaments depolymerizing and inducing motor unbinding, and ACP unbinding. Thus x is a ramp function starting at $t = 0$:

$$t < t_1 : x(t) = v * t \qquad \text{(S6.9)}$$

$$t \geq t_1 : x(t) = x_1 = v * t_1 \qquad \text{(S6.10)}$$

where v is the rate of deformation of the network due to motors walking. v is thus proportional to the average walking speed of motors and $\frac{C_{\mathrm{M}}}{C_{\mathrm{tot}}}$. t_1 depends on the turnover rate of the network, as further deformations are prevented as motors or ACPs become unbound. For simplicity, we assume that t_1 takes the form:

$$t_1 = \frac{1}{k_{\mathrm{u}}} \qquad \text{(S6.11)}$$

which indicates the persistence (or processivity) time of motors, empirically estimated to be on the order of 10 seconds in live cells [38].

Taking everything together, we can derive a constitutive relation for the time evolution of the forces exhibited in the active kinetic spring model:

$$F(t) = \frac{K_0}{k_\mathrm{b}+k_\mathrm{u}}\left(k_\mathrm{r} + k_\mathrm{u}\mathrm{e}^{-(\mathrm{k_b}+\mathrm{k_u})t}\right) * x(t) \qquad \text{(S6.12)}$$

where $K_0 = qC_{tot}$ is the spring constant of the network at $t = 0$, and recall that $x(t)$ scales as $\frac{C_M}{C_{tot}}$. As shown in Figs. 6b-d, by tuning actin turnover rates and concentrations of motors and ACPs (springs), we can reproduce similar stress profiles and phase maps compared to those measured from Brownian dynamics simulations. Increasing C_M leads to larger peak stresses and lower sustainability, whereas increasing actin turnover or spring concentration (C_{tot}) stabilizes stress profiles. Equations S6.1 to S6.12 help provide insights toward the underlying parameters and scales that govern the relations between the kinetics and mechanics in the active cytoskeletal network. Note that since we are able to capture long-term network behaviors with reduced computational cost, we can see the emergence of metastable states in which unstable configurations do not dissipate forces completely as shown in Fig. 6b, which may correspond to superstructure formation such as stress fibers. S also has to be calculated differently due to these states, so we determined S here by calculating the ratio of the long-time force to the peak force (rather than calculating the decay rate of the force).

Supplementary References

1 Jung, W., Murrell, M. P. & Kim, T. F-actin cross-linking enhances the stability of force generation in disordered actomyosin networks. *Comp Part Mech*, online first (2015).

2 Kishino, A. & Yanagida, T. Force measurements by micromanipulation of a single actin filament by glass needles. *Nature* **334**, 74-76 (1988).

3 Isambert, H. *et al.* Flexibility of actin filaments derived from thermal fluctuations. Effect of bound nucleotide, phalloidin, and muscle regulatory proteins. *The Journal of biological chemistry* **270**, 11437-11444 (1995).

4 Meyer, R. K. & Aebi, U. Bundling of actin filaments by alpha-actinin depends on its molecular length. *J Cell Biol* **110**, 2013-2024 (1990).

5 Morel, J. E., Bachouchi-Salhi, N. & Merah, Z. Shape and length of myosin heads. *Journal of theoretical biology* **156**, 73-90 (1992).

6 Uyeda, T. Q., Abramson, P. D. & Spudich, J. A. The neck region of the myosin motor domain acts as a lever arm to generate movement. *Proceedings of the National Academy of Sciences of the United States of America* **93**, 4459-4464 (1996).

7 Ferrer, J. M. *et al.* Measuring molecular rupture forces between single actin filaments and actin-binding proteins. *Proceedings of the National Academy of Sciences* **105**, 9221-9226 (2008).

8 Erdmann, T., Albert, P. J. & Schwarz, U. S. Stochastic dynamics of small ensembles of non-processive molecular motors: the parallel cluster model. *The Journal of chemical physics* **139**, 175104 (2013).

9 Erdmann, T. & Schwarz, U. S. Stochastic Force Generation by Small Ensembles of Myosin II Motors. *Physical Review Letters* **108**, 188101 (2012).

10 Craig, R. & Megerman, J. Assembly of smooth muscle myosin into side-polar filaments. *J Cell Biol* **75**, 990-996 (1977).

11 Clift, R., Grace, J. R. & Weber, M. E. *Bubbles, drops, and particles*. (Dover Publications, 2005).

12 Liu, X. & Pollack, G. H. Mechanics of F-actin characterized with microfabricated cantilevers. *Biophys J* **83**, 2705-2715 (2002).

13 Kim, T., Hwang, W., Lee, H. & Kamm, R. D. Computational analysis of viscoelastic properties of crosslinked actin networks. *PLoS Comput Biol* **5**, e1000439 (2009).

14 Veigel, C., Molloy, J. E., Schmitz, S. & Kendrick-Jones, J. Load-dependent kinetics of force production by smooth muscle myosin measured with optical tweezers. *Nat Cell Biol* **5**, 980-986 (2003).

15 Uemura, S., Higuchi, H., Olivares, A. O., De La Cruz, E. M. & Ishiwata, S. Mechanochemical coupling of two substeps in a single myosin V motor. *Nature structural & molecular biology* **11**, 877-883 (2004).

16 Kim, T. Determinants of contractile forces generated in disorganized actomyosin bundles. *Biomech Model Mechanobiol* **14**, 345-355 (2015).

17 Tyska, M. J. *et al.* Two heads of myosin are better than one for generating force and motion. *Proceedings of the National Academy of Sciences of the United States of America* **96**, 4402-4407 (1999).

18 McCullough, Brannon R. *et al.* Cofilin-Linked Changes in Actin Filament Flexibility Promote Severing. *Biophysical Journal* **101**, 151-159 (2011).

19 Pollard, T. D. Regulation of Actin Filament Assembly by Arp2/3 Complex and Formins. *Annual Review of Biophysics and Biomolecular Structure* **36**, 451-477 (2007).

20 Romero, S. *et al.* Formin Is a Processive Motor that Requires Profilin to Accelerate Actin Assembly and Associated ATP Hydrolysis. *Cell* **119**, 419-429 (2004).

21 Pollard, T. D. & Borisy, G. G. Cellular Motility Driven by Assembly and Disassembly of Actin Filaments. *Cell* **112**, 453-465 (2003).

22 Carlier, M.-F. *et al.* Actin Depolymerizing Factor (ADF/Cofilin) Enhances the Rate of Filament Turnover: Implication in Actin-based Motility. *The Journal of Cell Biology* **136**, 1307-1322 (1997).

23 Bugyi, B. & Carlier, M.-F. Control of Actin Filament Treadmilling in Cell Motility. *Annual Review of Biophysics* **39**, 449-470 (2010).

24 Andrianantoandro, E. & Pollard, T. D. Mechanism of Actin Filament Turnover by Severing and Nucleation at Different Concentrations of ADF/Cofilin. *Molecular Cell* **24**, 13-23 (2006).

25 McCullough, B. R., Blanchoin, L., Martiel, J.-L. & De La Cruz, E. M. Cofilin Increases the Bending Flexibility of Actin Filaments: Implications for Severing and Cell Mechanics.

Journal of Molecular Biology **381**, 550-558 (2008).

26 Bear, J. E. *et al.* Antagonism between Ena/VASP Proteins and Actin Filament Capping Regulates Fibroblast Motility. *Cell* **109**, 509-521 (2002).

27 Hartwig, J. H. & Shevlin, P. The architecture of actin filaments and the ultrastructural location of actin-binding protein in the periphery of lung macrophages. *The Journal of Cell Biology* **103**, 1007-1020 (1986).

28 Podolski, J. L. & Steck, T. L. Length distribution of F-actin in Dictyostelium discoideum. *Journal of Biological Chemistry* **265**, 1312-1318 (1990).

29 Kasza, K. E. *et al.* Actin Filament Length Tunes Elasticity of Flexibly Cross-Linked Actin Networks. *Biophysical Journal* **99**, 1091-1100 (2010).

30 Koenderink, G. H. *et al.* An active biopolymer network controlled by molecular motors. *Proceedings of the National Academy of Sciences* **106**, 15192-15197 (2009).

31 Li, A. *et al.* The Actin-Bundling Protein Fascin Stabilizes Actin in Invadopodia and Potentiates Protrusive Invasion. *Current Biology* **20**, 339-345 (2010).

32 Shin, J. H., Gardel, M. L., Mahadevan, L., Matsudaira, P. & Weitz, D. A. Relating microstructure to rheology of a bundled and cross-linked F-actin network in vitro. *Proceedings of the National Academy of Sciences* **101**, 9636-9641 (2004).

33 Hoffman, B. D., Grashoff, C. & Schwartz, M. A. Dynamic molecular processes mediate cellular mechanotransduction. *Nature* **475**, 316-323 (2011).

34 Murrell, M. P. & Gardel, M. L. F-actin buckling coordinates contractility and severing in a biomimetic actomyosin cortex. *Proceedings of the National Academy of Sciences* **109**, 20820-20825 (2012).

35 Wilson, C. A. *et al.* Myosin II contributes to cell-scale actin network treadmilling through network disassembly. *Nature* **465**, 373-377 (2010).

36 Haviv, L., Gillo, D., Backouche, F. & Bernheim-Groswasser, A. A Cytoskeletal Demolition Worker: Myosin II Acts as an Actin Depolymerization Agent. *Journal of Molecular Biology* **375**, 325-330 (2008).

37 Reymann, A.-C. *et al.* Actin Network Architecture Can Determine Myosin Motor Activity. *Science* **336**, 1310-1314 (2012).

38 Guo, M. *et al.* Probing the Stochastic, Motor-Driven Properties of the Cytoplasm Using

Force Spectrum Microscopy. *Cell* **158**, 822-832 (2014).

39 Mak, M., Kamm, R. D. & Zaman, M. H. Impact of Dimensionality and Network Disruption on Microrheology of Cancer Cells in 3D Environments. *PLoS Comput Biol* **10**, e1003959 (2014).

40 Lau, A. W. C., Hoffman, B. D., Davies, A., Crocker, J. C. & Lubensky, T. C. Microrheology, Stress Fluctuations, and Active Behavior of Living Cells. *Physical Review Letters* **91**, 198101 (2003).

41 Mizuno, D., Tardin, C., Schmidt, C. F. & MacKintosh, F. C. Nonequilibrium Mechanics of Active Cytoskeletal Networks. *Science* **315**, 370-373 (2007).

42 Hoffman, B. D., Massiera, G., Van Citters, K. M. & Crocker, J. C. The consensus mechanics of cultured mammalian cells. *Proceedings of the National Academy of Sciences* **103**, 10259-10264 (2006).

43 Saks, V., Beraud, N. & Wallimann, T. Metabolic Compartmentation – A System Level Property of Muscle Cells. *International Journal of Molecular Sciences* **9**, 751-767 (2008).

44 Szymanski, J. & Weiss, M. Elucidating the Origin of Anomalous Diffusion in Crowded Fluids. *Physical Review Letters* **103**, 038102 (2009).

45 Zhou, H.-X., Rivas, G. & Minton, A. P. Macromolecular Crowding and Confinement: Biochemical, Biophysical, and Potential Physiological Consequences*. *Annual Review of Biophysics* **37**, 375-397 (2008).

46 Luby-Phelps, K. The physical chemistry of cytoplasm and its influence on cell function: an update. *Molecular biology of the cell* **24**, 2593-2596 (2013).

47 Nakamura, F., Stossel, T. P. & Hartwig, J. H. The filamins. *Cell Adhesion & Migration* **5**, 160-169 (2011).

48 Panorchan, P., Lee, J. S. H., Kole, T. P., Tseng, Y. & Wirtz, D. Microrheology and ROCK Signaling of Human Endothelial Cells Embedded in a 3D Matrix. *Biophysical Journal* **91**, 3499-3507 (2006).

49 Bell, G. Models for the specific adhesion of cells to cells. *Science* **200**, 618-627 (1978).
